# Dual Effect of Bleomycin on Histopathological Features of Lungs and Mediastinal Fat-Associated Lymphoid Clusters in an Autoimmune Disease Mouse Model

**DOI:** 10.3389/fimmu.2021.665100

**Published:** 2021-07-21

**Authors:** Yaser Hosny Ali Elewa, Osamu Ichii, Teppei Nakamura, Yasuhiro Kon

**Affiliations:** ^1^ Department of Histology and Cytology, Faculty of Veterinary Medicine, Zagazig University, Zagazig, Egypt; ^2^ Laboratory of Anatomy, Department of Basic Veterinary Sciences, Faculty of Veterinary Medicine, Hokkaido University, Sapporo, Japan; ^3^ Laboratory of Agrobiomedical Science, Faculty of Agriculture, Hokkaido University, Sapporo, Japan; ^4^ Section of Biological Safety Research, Chitose Laboratory, Japan Food Research Laboratories, Hokkaido, Japan

**Keywords:** bleomycin, immune cell infiltration, lung injury score, mediastinal adipose tissue, high endothelial venules

## Abstract

Mediastinal fat-associated lymphoid clusters (MFALCs) are novel immune clusters that function in the pathogenesis of bleomycin (BLM)-induced pneumonitis in a C57BL/6 mouse model. However, we lack literature on the effects of BLM in an autoimmune disease mouse model (AIDM). In the present study, BLM sulfate (BLM group) or phosphate-buffered saline (PBS group) were intranasally administered in BXSB/MpJ-Yaa (Yaa) AIDM and its wild-type strains (BXSB/MpJ “BXSB”) and the histopathology of MFALCs and lungs were examined on days 7 and 21 days. Immunohistochemical analysis was performed to detect lymphatic vessels (LVs), high endothelial venules (HEVs), proliferating, and immune cells. Furthermore, the mRNA expression of Yaa locus genes (*TLR7*, *TLR8*, *Arhgap6*, *Msl3*, and *Tceanc*) was detected in the lung tissues. Here, we show a dual effect of BLM on intra-thoracic immune hemostasis among Yaa AIDM and its corresponding wild-type strain (BXSB mice). The BLM group of BXSB mice displayed significantly higher values of lung injury scores (LIS) and size of MFALCs as compared with the corresponding PBS group. However, an opposite effect was detected in Yaa mice. Furthermore, Yaa mice displayed decreased serum autoantibody titers and downregulated expression of *TLR7*, *TLR8*, *Msl3*, and *Tceanc* in the lungs following BLM administration, especially on day 21. Interestingly, significant positive correlations were detected in both strains between the LIS and the size of MFALCs, LVs, HEVs, and proliferating cells. Conclusively, our findings revealed a crucial function of HEVs on the extent of lung injury and the development of MFALCs in BLM-administered Yaa AIDM and control BXSB mice with dual effects. Moreover, our data suggest that down regulation of Yaa locus genes could contribute as an important attributing factor leading to decrease in the degree of autoimmunity and lung injury in AIDM. Therefore, we suggest that genetic background contributes to BLM diversity among AIDM and the wild-type strain. Targeting some genes or venules could provide novel therapeutic approaches for some autoimmune-associated respiratory diseases *via* controlling the MFALCs development.

## Introduction

Fat-associated lymphoid clusters (FALCs) are atypical lymphoid clusters (LCs) associated with adipose tissues on various mucosal surfaces, including the mediastinum, pericardium, mesenteries, omentum, and gonadal fat in humans and mice (1–4). FALCs were first identified in healthy mouse and human mediastinal and mesenteric adipose tissues and consist of numerous macrophages, T and B lymphocytes, as well as a small number of granulocytes. Moreover, these are enriched with blood vessels, lymphatic vessels (LVs), and high endothelial venules (HEVs) (1, 2, 5). FALCs have been recently implicated in the progression of several diseases in both mice and humans and in protecting against helminth infections (1). In addition, mesenteric FALCs markedly increase in number and size on day 3 following zymosan-induced peritoneal inflammation (3). Furthermore, our previous studies have suggested a possible function of mediastinal FALCs (MFALCs) in the pathogenesis of lung injuries in both autoimmune mouse model (MRL/MpJ-*lpr* mice “Lpr” and BXSB/MpJ-*Yaa “*Yaa*”* mice) (6) and bleomycin (BLM)-induced pneumonitis C57BL/6 (B6) mice model (7).

BLM is an antitumor drug that has been used successfully to treat a variety of malignancies, including Hodgkin’s lymphoma that usually affects young individuals and germ cell testicular tumors. However, its therapeutic use in humans is associated with the development of pulmonary toxicity and fibrosis in up to 10% of patients receiving it (8). Similarly, differences in the susceptibility to fibrosis of mouse strains following administration of a single dose of BLM have been reported in which C57BL/6 (B6) mice were susceptible to BLM-induced lung injury and developed pneumonitis and lung fibrosis (7, 9). In contrast, other strains, such as DBA/2 (DBA), BALB/c, and C3H/HeJ mice, are resistant to BLM-induced lung injury and fibrosis (10, 11). Interestingly, our previous study revealed a difference in the size of MFALCs between different strains in which the DBA mice showed less developed MFALCs than B6 mice (2). Furthermore, we have recently reported a dramatic increase in the size of MFALCs following administration of a single dose of BLM in B6 mice at both 7 and 21 days. In addition, we have reported a significant positive correlation between the size of MFALCs and lung injury following BLM administration, suggesting the involvement of MFALCs in the pathogenesis of BLM-induced fibrosis (7). Although genetic susceptibility is an important risk factor for several diseases as well as susceptibility to BLM-induced fibrotic lung injury (10), the effect of BLM on both lung injury and size of MFALCs in both the autoimmune disease mouse model (that showed severe lung injury and well-developed MFALCs) and its control strains (that showed normal lung architecture and less developed MFALCs) (6) has not yet been established.

Numerous autoimmune murine models are currently being used to understand the cellular and genetic aspects of the progression of autoimmune disease in humans, such as Lpr and Yaa, along with a comparison with their healthy control mice MRL/MpJ “MpJ” and BXSB/MpJ “BXSB,”, respectively (12). Such autoimmune mouse models develop lupus-like diseases with symptoms similar to those of human systemic lupus erythematosus (SLE) (13). The autoimmune lesions in the Lpr mouse developed in both sexes and resulted from a mutation in a single gene (Fas receptor gene) (14), and defects in this gene resulted in marked lymphadenopathy and splenomegaly (15). In contrast, the autoimmune disease occurred only in male Yaa mice due to the translocation of an X chromosomal region onto the Y chromosome (16), resulting in 15 duplicated genes on the Y chromosome (17).

Interestingly, we have recently reported high expression of certain genes on Yaa locus, such as Toll-like receptor 7 (*Tlr7*) and *Tlr8*, in Yaa mice than in BXSB mice at both young (three months) and old (six months) ages. Moreover, the expression of other Yaa locus genes, such as male-specific lethal-subunit 3 (*Msl3*), Rho GTPase activating protein 6 (*Arhgap6*), and transcription elongation factor A (SII) N-terminal (*Tceanc)* was significantly increased with the progression of autoimmune disease (18).

The genetic basis of susceptibility to BLM-induced pulmonary fibrosis in several healthy strains has been established (10). Our previous report in genetically mutated autoimmune mouse models (Lpr and Yaa) indicated more developed MFALCs and severe lung injury in comparison with the control strains (MpJ and BXSB) (6). However, it is still unknown whether such genetic mutations in autoimmune mouse models could affect their response to BLM. Therefore, in the present study, we used the autoimmune disease model strain (Yaa) and their control strain (BXSB) to study the genetic variations regulating the response of BLM-induced pulmonary fibrosis.

## Materials And Methods

### Ethics Statement

The animal experiments were performed under a protocol approved by the Institutional Animal Care and Use Committee of the Graduate School of Veterinary Medicine, Hokkaido University (approval no. 15–0079).

### Experimental Animals and Design

We used the autoimmune mouse model established in adult Yaa male mice of 12 weeks of age and its control strain (BXSB mice). Mice were purchased from Japan SLC, Inc. (Shizuoka, Japan) and housed in a specific pathogen-free facility with free access to water and chow. Sixteen mice belonging to each strain were used and divided into two groups, including BLM and phosphate-buffered saline (PBS). In the BLM group, 50 µL of BLM sulfate (LKT Laboratories, Inc., catalog number: B4518, lot number: 2599253) was diluted in sterile PBS at a concentration of 5 mg/kg, was administered as a single intranasal dose. The control group received 50 µL of sterile PBS intranasally. on days 7 and 21, four mice from each group were subjected to deep anesthesia using a mixture of medetomidine (0.3 mg/kg), butorphanol (5.0 mg/kg), and midazolam (4.0 mg/kg). At the same time, the blood was collected by cutting of femoral artery, and transcardial perfusion with PBS was performed. Then the spleens were harvested from mice and weighted in all groups, and both mediastinal fat tissues (MFTs) and right lung lobes were immediately collected and fixed in 4% paraformaldehyde overnight at 4°C. The left lung lobe from different groups was immediately inserted into liquid nitrogen and stored at –80°C until further analysis.

### Tissue Preparation for Histopathological Analysis

The specimens were washed following overnight fixation, dehydrated in ascending grades (70, 80, 90, 95%, absolute I, II, and III) of alcohol, cleared in xylene, and subsequently embedded in paraffin. Paraffin sections (3 µm) of MFTs were prepared for histopathological analysis. Sections were stained with H&E and Masson’s trichrome (MT). Furthermore, immunohistochemistry was performed using 3 µm thick sections to detect different vessels (PNAd^+^ HEVs and LYVE-1^+^ LVs), immune cells (B220^+^ B cells, CD3^+^ T cells, Iba1^+^ macrophages, and Gr1^+^ granulocytes), and BrdU^+^ proliferating cells. BrdU-incorporating cells were detected as described in our previous study (2). Immunohistochemical staining was performed following the method described in our previous study (19). The details of antigen retrieval and optimal primary antibody dilutions are summarized in **Table 1**. Additionally, the lung sections of different studied groups were stained with rabbit polyclonal anti-collagen I antibody, and anti-COL3A1 to detect degree of collagen depositions for different collagen types according to our recent report (20). Furthermore, double immunofluorescence staining was performed to reveal the occurrence of B220^+^ B cells and CD3^+^ T cells in both MFALCs and the lungs of different groups in both studied strains. The immunofluorescent images were captured by a fluorescence microscope (BZX-710; Keyence; Osaka, Japan).

**Table 1:** List of antibodies, working dilutions, and methods for antigen retrieval.

Antibody	Source	Dilution	Antigen retrieval	Heating condition
Rat anti-BrdU	Abcam (Tokyo, Japan)	1:200	10 mM citrate buffer (pH 6.0)	105°C, 20 min
Rabbit anti-CD3	Nichirei (Tokyo, Japan)	1:200	10 mM Tris-HCl buffer (pH 7.4)	105°C, 20 min
Rat anti-B220	Cedarlane (Ontario, Canada)	1:1600	10 mM citrate buffer (pH 6.0)	105°C, 20 min
Rabbit anti-Iba1	Wako (Osaka, Japan)	1:1200	10 mM citrate buffer (pH 6.0)	105°C, 20 min
Rat anti-Gr1	R and D system (Minneapolis, USA)	1:800	0.1% pepsin/0.2 N HCl	37°C, 5 min
Rabbit anti-LYVE-1	Adipogen (San Diego, CA, USA)	1:500	10 mM citrate buffer (pH 6.0)	105°C, 20 min
Rat anti-PNAd	BioLegend (San Diego, CA, USA)	1:500	10 mM Tris-HCl buffer (pH 7.4)	105°C, 20 min

### Quantitative Polymerase Chain Reaction Analysis

Total RNA was purified from the frozen lung samples of each group using TRIzol reagent (Thermo Fisher Scientific; Waltham, MA, USA) according to the manufacturer’s instructions. The purified total RNA of each sample was used as a template to synthesize cDNA using ReverTra Ace qPCR RT Master Mix (Toyobo Co., Osaka, Japan). Thereafter, the cDNA (20 ng/µL) from each sample was used for qPCR analysis using either TaqMan PCR method (for analysis of fibrosis genes: actin, alpha 2, smooth muscle, aorta [*Acta2*]; collagen, type I, alpha 1 [*Col1a1*]; and collagen, type III, alpha 1 [*Col3a1*]) as we previously described (7) or THUNDERBIRD^®^ SYBR^®^ qPCR Mix (Toyobo Co., Ltd.) for the analysis of Yaa locus genes (*TLR7*, *TLR8*, *Arhgap6*, *Msl3*, and *Tceanc*) as we recently reported (18). The gene expression data were normalized using the housekeeping β-actin (*ACTB*) gene.

### Enzyme-Linked Immunosorbent Assay for Serum Autoantibody Measurement

To evaluate the extent of systemic autoimmune conditions, the titers of anti-dsDNA autoantibodies in the sera of mice were measured by enzyme-linked immunosorbent assay (ELISA) using the Alpha Diagnostic International Inc. mouse anti-dsDNA ELISA Kit (M-5110; San Antonio, TX, USA) according to the manufacturer’s instructions.

### Histomorphometric Measurements

For histoplanimetry, four mice were analyzed per group on day 7 (early time points) and day 21 (late time points). Using the BZ-X710 microscope (Keyence; Osaka, Japan), digital images from H&E, MT, and immune-stained tissue sections were captured for further analysis. The ratio of lymphoid clusters (LCs) area/total MFTs area was calculated from the H&E-stained digital images using the ImageJ software (ver. 1.32j, http://rsb.info.nih.gov/ij), as described in our previous reports (7, 20). Furthermore, the extent of lung injury was measured using images of MT-stained lung sections from grade 0 to 8 according to the scoring criteria reported by Ashcroft et al. (21), and the average of such scores per mouse was recorded. Briefly, different digital images from microscopic fields of all lung tissues for each mouse belonging to different groups were captured. Thereafter, we graded the field with normal lung structure as grade 0 and scored the field with lung lesions as grades 1 to 8. In grade 1, the lesions corresponded to a slight thickening of the alveolar wall with a few cellular infiltrations. In grade 3, the lesions were characterized by a slight thickening of the alveolar wall with moderate cellular infiltrations. In grade 5, numerous cellular infiltrations with mild fibrosis and damage to the lung architecture were observed. In grade 7, severe damage of lung tissue with clear fibrous masses was observed. Grade 8 showed total replacement of lung tissues with fibrous tissue. We used the odd number grade for scaling of the extent of lung injury; however, in case of difficulty in distinguishing between the two odd number grades, we used the intervening even-numbered grades. Subsequently, the scores of different microscopic fields/mice were recorded, and the average score of all grades was quantified. Furthermore, the lung injury scores of different groups from both studied strains (Yaa, and BXSB mice) were compared to that of photographs captured from MT-stained lung sections from the wild-type strain (B6 mice) treated with either BLM or PBS in our previous study at the indicated time points (**Supplementary Figure 1**).

For immune-stained MFTs and lung tissue sections, digital images were captured at ×200 power field using the BZ-X710 (Keyence, Osaka, Japan), and the following measurements were performed using the BZ-X analyzer (Keyence): percentage of positive area ratio of collagen I, and COL3A1 immuno-stained lung sections, relative area ratio of LYVE-1^+^ LVs in the lungs and MFALCs and PNAd^+^ HEVs in MFALCs (as previously described (7), PNAd^+^ HEVs number/mice in the lung field, and the positive area ratio of different immune cells (B and T lymphocytes, macrophages, and granulocytes) in both MFALCs and lung fields in different groups. To analyze the extent of proliferation within both MFALCs and lung lobes, the number of BrdU^+^ proliferative cells was counted and represented as the percentage of either cell density ratio in MFALCs (number of positive cells divided by the total number of cells within the clusters) or as a positive index ratio in lung (number of positive cells divided by the field area of different lung fields). Such percentages were compared between the two groups. Furthermore, we examined the spleen/body weight (B.W.) ratios.

### Statistical Analysis

Differences between the groups were compared using the Kruskal–Wallis test, followed by Scheffé’s method for multiple comparisons when significant differences were observed. Numerical data are presented as mean± standard error. *P*-values < 0.05 were considered statistically significant. To analyze the correlation between two variables, Spearman’s correlation test was used (^*^significant value, *P* < 0.05, ^**^highly significant value, *P* < 0.01).

## Results

### Morphological Features of MFALCs in BLM Mouse Model Versus the Phosphate-Buffered Saline Control Group

**Figure 1:**
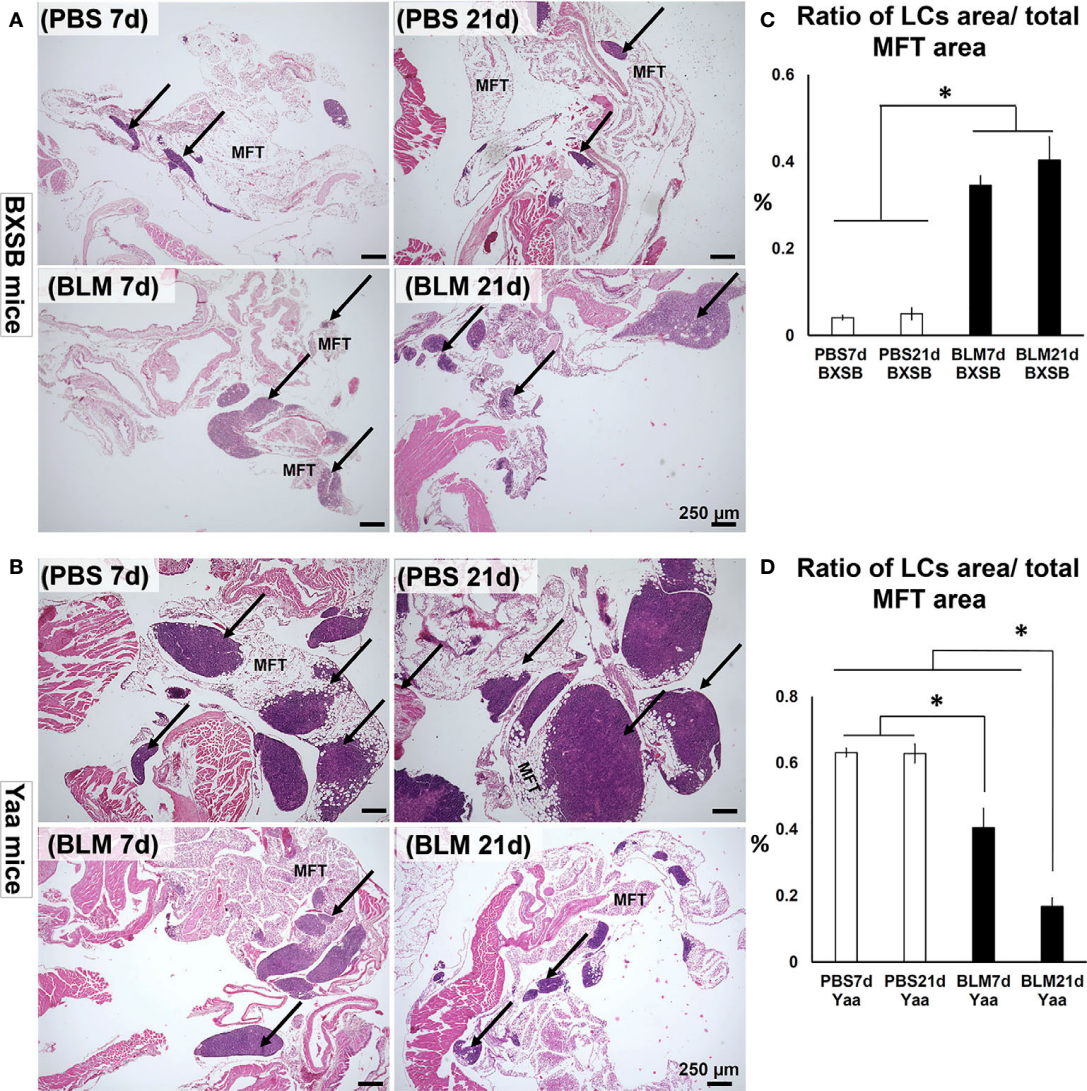
Degree of mediastinal fat-associated lymphoid cluster (MFALC) development in BLM and PBS groups of both autoimmune disease mice model (Yaa) and their wild-type strain (BXSB) on days 7 and 21. **(A, B)** Representative histopathological images of H&E stained mediastinal fat tissue (MFT) sections in both BXSB **(A)** and Yaa **(B)**. Arrows indicate MFALCs. **(C, D)** Graphs showing the percentages of the ratio of lymphoid clusters (LCs) area/total mediastinal fat tissue (MFT) area in control strain “BXSB” **(C)** and its autoimmune disease mouse model “Yaa” **(D)**. Asterisk indicates significant differences between PBS and BLM groups, analyzed by the Kruskal–Wallis test, and followed by Scheffé’s method. (*P* < 0.05); *n* = 4 in each experimental group. Values are expressed as mean ± standard error (SE).

Examination of hematoxylin and eosin (H&E)-stained sections of MFTs in BXSB mice revealed more developed MFALCs in the BLM groups than in the PBS groups at both time points (days 7 and 21) (**Figure 1A**). However, a remarkable decrease in such clusters was observed in Yaa mice following BLM administration when compared with the PBS groups on days 7 and 21 (**Figure 1B**). To assess the histological index of development of MFALCs, we measured the area ratio of LCs/MFTs in H&E-stained sections. As shown in **Figure 1C**, the sizes of MFALCs were significantly larger in the BLM group of BXSB mice at both time points in comparison with those in the PBS-treated control group. In contrast, a significant decrease in the LCs/MFTs area ratios in the BLM group of Yaa mice was observed on both 7 and 21 days than in the PBS group. Furthermore, such ratios were significantly lower in the BLM group on day 21 than that on day 7 (**Figure 1D**).

### Histopathological Analysis of the Degree of Lung Injury in BLM and PBS-Treated Groups

**Figure 2:**
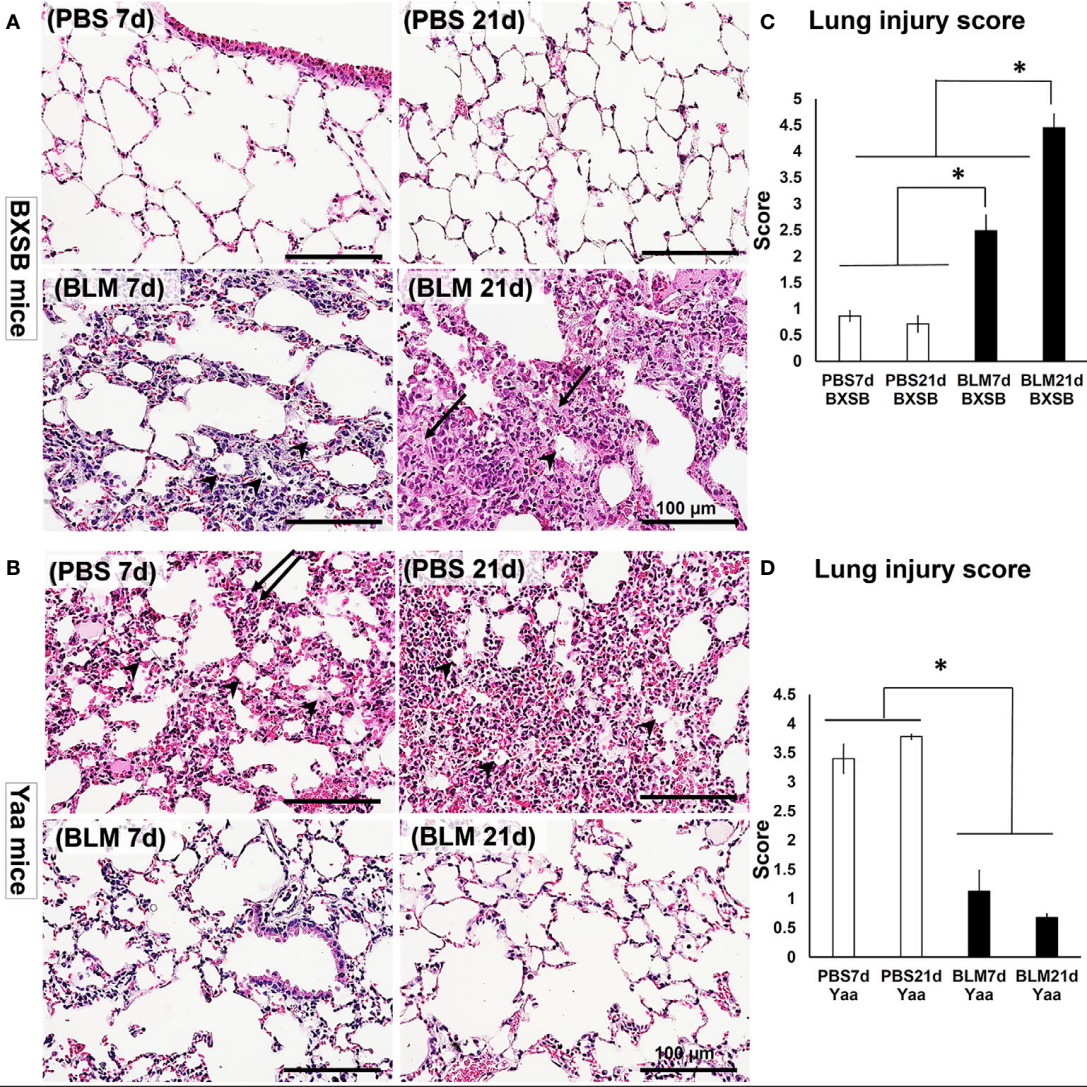
Degree of lung injury in BLM and PBS groups of both autoimmune disease mice model (Yaa) and their wild-type strain (BXSB) on days 7 and 21. **(A, B)** Representative histopathological images of H&E-stained lung tissue sections in both BXSB **(A)** and Yaa **(B)**. More connective tissue deposition in the parenchyma of BLM group in BXSB mice on day 21 (arrows), collapsed alveoli (arrow heads), and thickening of the inter-alveolar space (double arrows). **(C, D)** Graphs showing the average of lung injury score in control strain “BXSB” **(C)** and its autoimmune disease mouse model “Yaa” **(D)**. Asterisk indicates significant differences between PBS and BLM groups, analyzed by the Kruskal–Wallis test, and followed by Scheffé’s method. (*P* < 0.05); *n* = 4 in each experimental group. Values are expressed as mean ± standard error (SE).

H&E-stained lung tissue sections in PBS groups of BXSB mice (days 7 and 21) showed the normal histological structure of the lung tissues, including thin-walled alveoli with scarce mononuclear cellular infiltration. However, disruption of the normal lung architecture was observed in the BLM group. Examination of the lung sections of the BLM group on day 7 revealed high cellular infiltration in the interstitial tissue, thickening of the inter-alveolar space, and a few collapsed alveoli. On day 21, the BLM group showed severely distorted parenchyma with an increased disposition of connective tissue (CT), replacing the normal alveoli and numerous collapsed alveoli (**Figure 2A**).

As shown in **Figure 2B**, the lung tissues of the PBS group in Yaa mice showed numerous mononuclear cellular infiltrations into the lung parenchyma and several collapsed alveoli with thickening of the interalveolar septa on day 7. On day 21, the lung tissues in the PBS group showed severe lung lesions represented by high cellular infiltration that replaced most of the lung parenchyma with a few collapsed alveoli. Interestingly, moderate cellular infiltration with a slight thickening of the alveolar wall and interalveolar septum were observed following BLM administration in the lung tissues of Yaa mice on day 7. On day 21, the lung tissues in the BLM group of Yaa mice showed restoration of the normal lung architecture with little cellular infiltration and thin alveolar wall, suggesting the recovery of lung inflammation after BLM exposure.

To detect the extent of lung injury, we graded several MT-stained lung microscopic fields from PBS and BLM groups in both BXSB and Yaa mice on days 7 and 21 into scores according to the criteria described by Ashcroft et al. (22). The average of scores was compared between the groups in both the strains. Interestingly, the average lung injury score in BXSB mice increased significantly in the BLM group on days 7 and 21 than those of the PBS group. Furthermore, the BLM group showed significantly higher lung injury scores on day 21 than on day 7 (**Figure 2C**). In contrast, a sharp significant decrease in the lung injury scores in the BLM group of Yaa mice on both days 7 and 21 was observed than those in the PBS group (**Figure 2D**). As shown in **Supplementary Figure 1**, the BLM group of wild-type strain (B6 mice) at 21d showed significant higher lung injury score than other studied groups. On the other hand, the BLM group of B6 mice at 7d revealed significant difference in such score when compared with that of BXSB (BLM, and PBS groups at both 7 and 21 d), B6 (PBS groups at both 7 and 21 d), and Yaa (BLM groups at both 7 and 21 d) mice, but not with the PBS groups (7 and 21d) of Yaa mice.

### Extent of Lung Fibrosis in BLM and PBS-Treated Groups

**Figure 3:**
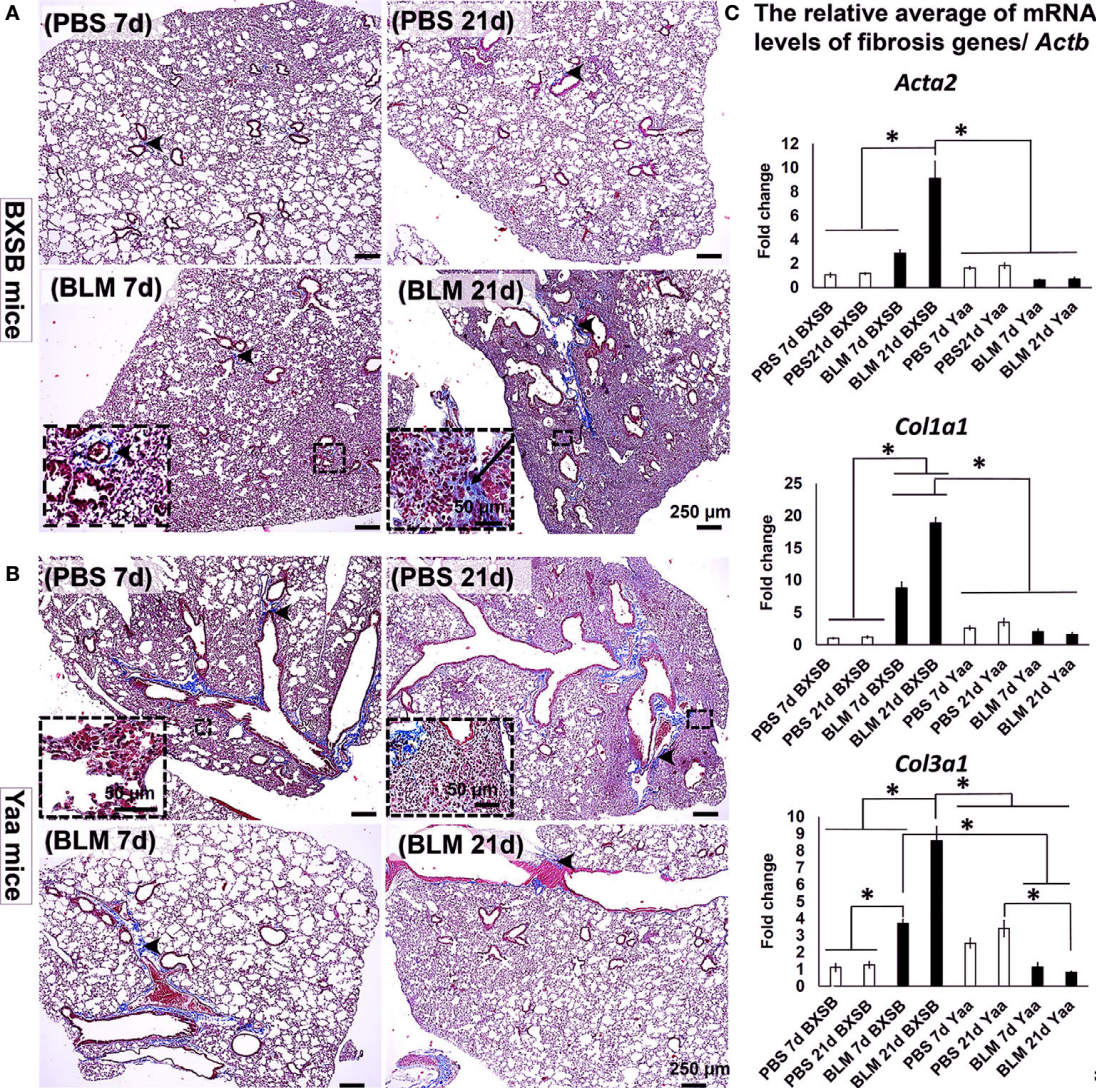
Degree of lung fibrosis in BLM and PBS groups of both autoimmune disease mice model (Yaa) and their wild-type strain (BXSB) on days 7 and 21. **(A, B)** Representative histopathological images of Masson’s trichrome (MT)-stained lung tissue sections in both BXSB **(A)** and Yaa **(B)**. C.T. Notice more aniline blue-positive deposition in the parenchyma of BLM group in BXSB mice on day 21 (arrows) and a few aniline blue-positive collagen fibers around the bronchioles (arrow heads). **(C)** Graphs showing the relative mRNA expression of candidate fibrosis genes (*Acta2, Col1a1*, and *Col3a1*) in the lung tissues of different groups in control strain “BXSB,” and its autoimmune disease mouse model “Yaa.” Asterisk indicates significant differences between the PBS and BLM groups, analyzed by the Kruskal–Wallis test, and followed by Scheffé’s method. (*P* < 0.05); *n* = 4 in each experimental group. Values are expressed as mean ± standard error (SE).

To identify the effect of BLM on the progression of lung inflammation and fibrosis in both BXSB and Yaa mice, we compared the histopathology of MT-stained lung sections between the PBS and BLM groups at both early and late time points (days 7 and 21, respectively). The PBS groups in BXSB mice showed a normal lung architecture with a few aniline blue-positive collagen fibers around the bronchioles. The BLM group showed high cellular infiltration and few aniline blue-positive collagen fibers around the bronchioles on day 7 and more aniline blue-positive collagen fiber depositions around the large bronchioles and within the lung parenchyma on day 21 (**Figure 3A**). The PBS groups in Yaa mice (on both days 7 and 21) showed high cellular infiltration and mild aniline blue-positive collagen fiber deposition around the bronchioles as well as within the parenchyma. However, the BLM groups at both time points showed a normal lung architecture with mild aniline blue-positive collagen fiber deposition around the large bronchioles (**Figure 3B**).

Interestingly, our previous studies have demonstrated significant upregulation of candidate fibrosis genes (*Acta2, Col1a1*, and *Col3a1*) in B6 mice at 21d following BLM administration (7). Therefore, to further explore the effects of BLM on the expression of such candidate fibrosis genes in autoimmune mouse model and their control strains, we compared the relative mRNA expression of these genes in the lung tissues of both BLM and PBS groups in Yaa and BXSB mice. The BLM group in BXSB mice showed significantly higher values for all studied fibrosis genes on day 21 than other BXSB and Yaa groups. Furthermore, the BLM administered-Yaa mice groups (on day 21) showed significantly reduced mRNA expression of *Col3a1* than the PBS-administered Yaa group on day 21 (**Figure 3C**). Similarly, immunohistochemical staining of lung sections with collagen I and COL3A1 antibodies among all studied groups revealed prominent collagen deposition in the lung sections of BLM group in BXSB mice at 21d than other studied groups (**Supplementary Figures 2A, B, D, E**). Morphometric measurement of the percentages of collagen I and COL3A1 positive area ratios revealed significant higher values than that of other groups in BXSB (**Supplementary Figure 2C**) and Yaa (**Supplementary Figure 2F**) mice. However, among the Yaa groups, no significant difference was observed among the PBS and BLM groups for collagen I positive area ratios at different time points (**Supplementary Figure 2C**). But for COL3A1 positive area ratios among Yaa studied groups, significant reduction was observed in the BLM group at both 7 and 21d than that of PBS at 21d (**Supplementary Figure 2F**).

### Analysis of Immune Cells in MFALCs and Lung Infiltration Between BLM-and PBS-Treated Groups

**Figure 4:**
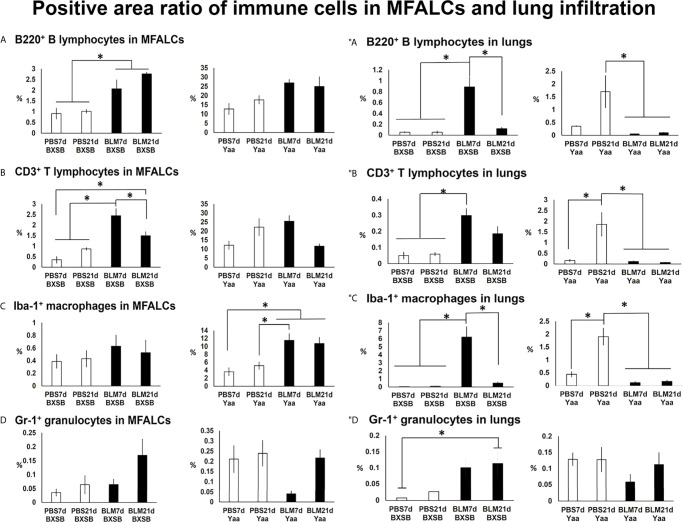
Analysis of immune cells within the MFALCs **(A–D)** and lungs **(″A–″D)** in BLM and PBS groups of both autoimmune disease mice model (Yaa) and their wild-type strain (BXSB) on days 7 and 21. **(A–D)** Graphs showing the positive area ratio of B220^+^ B cells **(A)**, CD3^+^ T cells **(B)**, Iba1^+^ macrophages **(C)**, and Gr1^+^ granulocytes **(D)** within MFALCs among different groups in both BXSB and Yaa mice. **(″A–″D)** Graphs showing the positive area ratio of B220^+^ B cells **(″A)**, CD3^+^ T cells **(″B)**, Iba1^+^ macrophages **(″C)**, and Gr1^+^ granulocytes **(″D)** within the lung tissue sections among different groups in both BXSB and Yaa mice. Asterisk indicates significant differences between PBS and BLM groups, analyzed by the Kruskal–Wallis test, followed by Scheffé’s method. (*P* < 0.05); *n* = 4 in each experimental group. Values are expressed as mean ± standard error (SE). Figures showing positive immune cells within MFALCs and lungs can be found in **Supplementay Figures 3**, **4**.

To analyze the changes in immune cell populations in MFALCs and lung infiltrates following BLM administration, immunohistochemical analysis was performed that detected B220^+^ B cells, CD3^+^ T cells, Iba1^+^ macrophages, and Gr1^+^ granulocytes. We compared the percentages of positive area ratios (PARs) for several immune cell populations within the MFALCs (**Supplementary Figures 3A, B**, **4A, B**) and lungs (**Supplementary Figures 3C, D**, **4C, D**) among different groups. Concerning the PARs for B220^+^ B cells and CD3^+^ T cells within the MFALCs of BXSB mice, the BLM groups showed significantly higher percentages than PBS groups (**Figures 4A, B** and **Supplementary Figure 3A**). Furthermore, a significantly reduced percentage of PAR of CD3^+^ T cells was observed in the BLM group on day 21 than on day 7 (**Figure 4B**). Also, the BLM group in Yaa mice showed a similar pattern of change for the percentage of PAR in both B220^+^ B cells and CD3^+^ T cells; however, there were no significant differences (**Figures 4A, B** and **Supplementary Figure 3A**). For the percentage of PAR of Iba1^+^ macrophages within the MFALCs, the BLM group in the BXSB and Yaa mice showed a high percentage than the control group. Such increase was non-significant in BXSB mice. However, significant values were observed in Yaa mice between the BLM groups (on days 7 and 21) and the PBS group (on day 7) and between the BLM groups (day 7) and the PBS group (day 21) (**Figure 4C** and **Supplementary Figures 4A, B**). In contrast, no significant difference could be observed in the percentage of PAR of Gr1^+^ granulocytes within the MFALCs among the groups in both the studied strains (**Figure 4D**).

With respect to infiltration of immune cells into the lungs (**Figures 4″A–″D**), the BLM group in BXSB mice showed a significant increase in the percentage of PAR of B220^+^ B cells, CD3^+^ T cells, and Iba1^+^ macrophages on day 7 than PBS groups (days 7 and 21). Furthermore, the BLM group on day 21 showed a decrease in the percentage of PAR of such immune cells than that of the BLM group on day 7. Such a decrease was significant for the percentages of PAR of B220^+^ B cells and Iba1^+^ macrophages (**Figures 4″A–″C** and **Supplementary Figures 3C**, **4C**). For Gr1^+^ granulocytes within the lungs, the BLM group showed a high percentage of PAR than the PBS groups. Such an increase was significant between the BLM group on day 21 and the PBS group on day 7 (**Figure 4″D**). On the contrary, a decrease in the percentages of PAR for the studied immune cell infiltration was observed in the BLM groups in the lungs of Yaa mice (on days 7 and 21) than in the PBS group on day 21. Such a decrease was significant for B220^+^ B cells, CD3^+^ T cells, and Iba1^+^ macrophages but not for Gr1^+^ granulocytes (**Figures 4″A–″D** and **Supplementary Figures 3D**, **4D**).

### Role of Peripheral Node Addressin^+^ HEVs in the Development of Lung Injury and MFALCs

**Figure 5:**
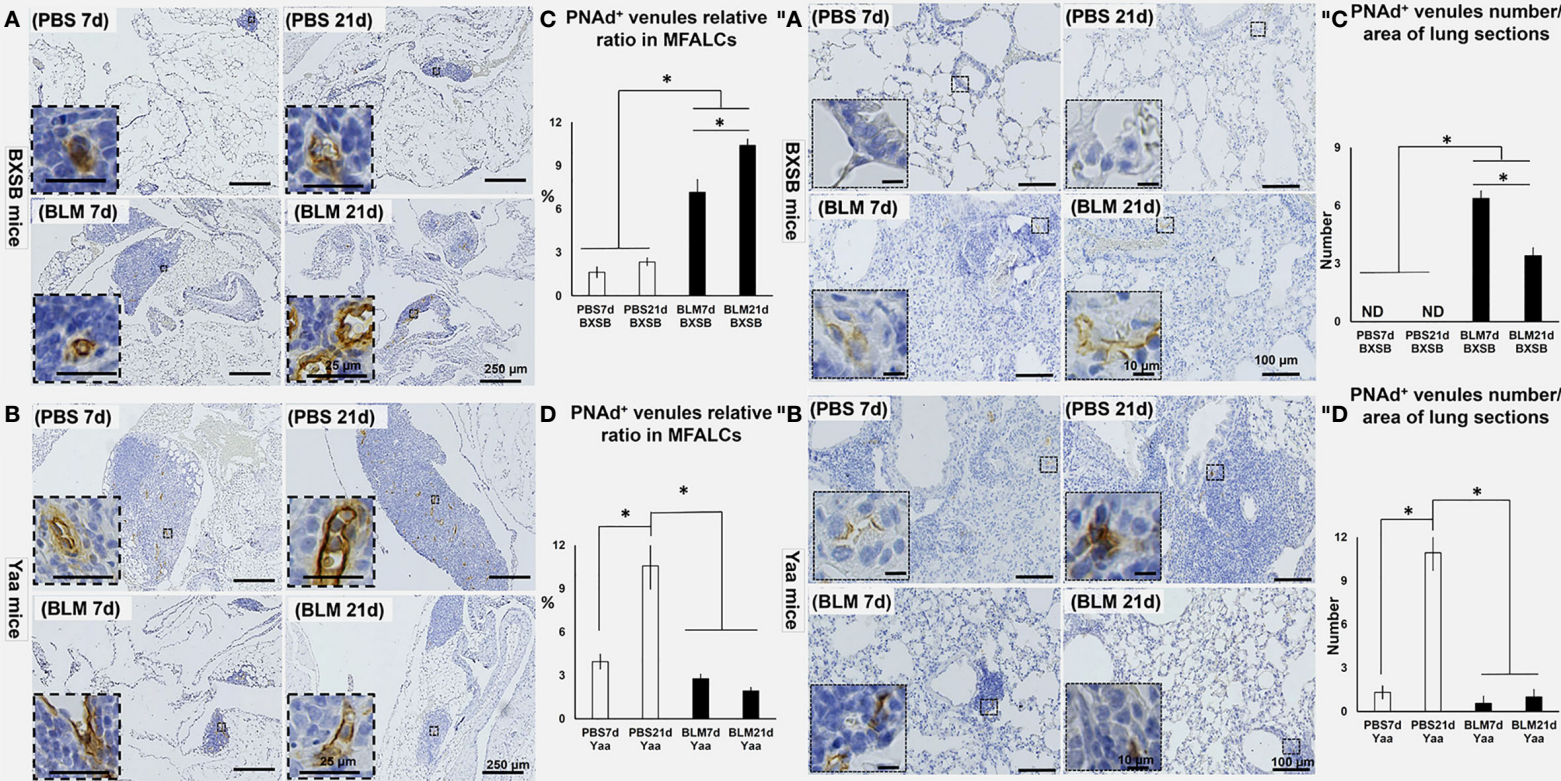
Analysis of the degree of PNAd^+^ HEVs development in MFALCs **(A–D)** and lungs **(″A–″D)** of BLM and PBS groups of both autoimmune disease mice model (Yaa) and their wild-type strain (BXSB) on days 7 and 21. **(A, B)** Representative histopathological images of immunohistochemically stained MFT sections with anti-PNAd antibody in both BXSB **(A)** and Yaa **(B)**. **(C, D)** Graphs showing the percentages of relative ratios of PNAd^+^ HEVs in MFALCs of control strain “BXSB” **(C)** and its autoimmune disease mouse models “Yaa” **(D)**. **(″A, ″B)** Representative histopathological images of immunohistochemically stained lung tissue sections with anti-PNAd antibody in both BXSB **(″A)** and Yaa **(″B)**. **(″C, ″D)** Graphs showing the average of PNAd^+^ HEVs number/lung field area of control strain “BXSB” **(″C)** and its autoimmune disease mouse models “Yaa” **(″D)**. Asterisk indicates significant differences between the PBS and BLM groups, analyzed by the Kruskal–Wallis test, followed by Scheffé’s method. (*P* < 0.05); *n* = 4 in each experimental group. Values are expressed as mean ± standard error (SE).

Our previous studies have revealed a possible function of HEVs in the development of MFALCs and progression of pneumonitis following BLM administration in B6 mice (7). In BXSB mice, highly developed PNAd^+^ HEVs were observed in MFALCs following BLM administration (**Figure 5A**). Furthermore, well-developed HEVs were observed in the lungs of BLM groups (on days 7 and 21); however, they were not detected in the PBS groups (**Figure 5″A**). Similarly, the morphometric measurements of relative ratios of PNAd^+^ HEVs in MFALCs and their numbers in the lungs revealed a significant increase following BLM administration. Furthermore, the BLM group on day 21 showed a significant increase in the relative ratios of PNAd^+^ HEVs in MFALCs and a significant decrease in their numbers in the lungs of the BLM group on day 7 (**Figures 5C, ″C**). In contrast, less developed HEVs were observed in MFALCs and lung tissues of the BLM group than that of the PBS group in Yaa mice (**Figures 5B, ″B**). Interestingly, a significant decrease in the relative ratios of PNAd^+^ HEVs in MFALCs and their numbers in the lungs was observed in the BLM group of Yaa mice on days 7 and 21 than in the PBS group. Furthermore, the PBS group showed a significant increase in the relative ratios of PNAd^+^ HEVs in MFALCs and their numbers in the lungs on day 21 than the PBS group on day 7 (**Figures 5D, ″D**).

### Analysis of LV Occurrence in Both MFALCs and Lungs

**Figure 6:**
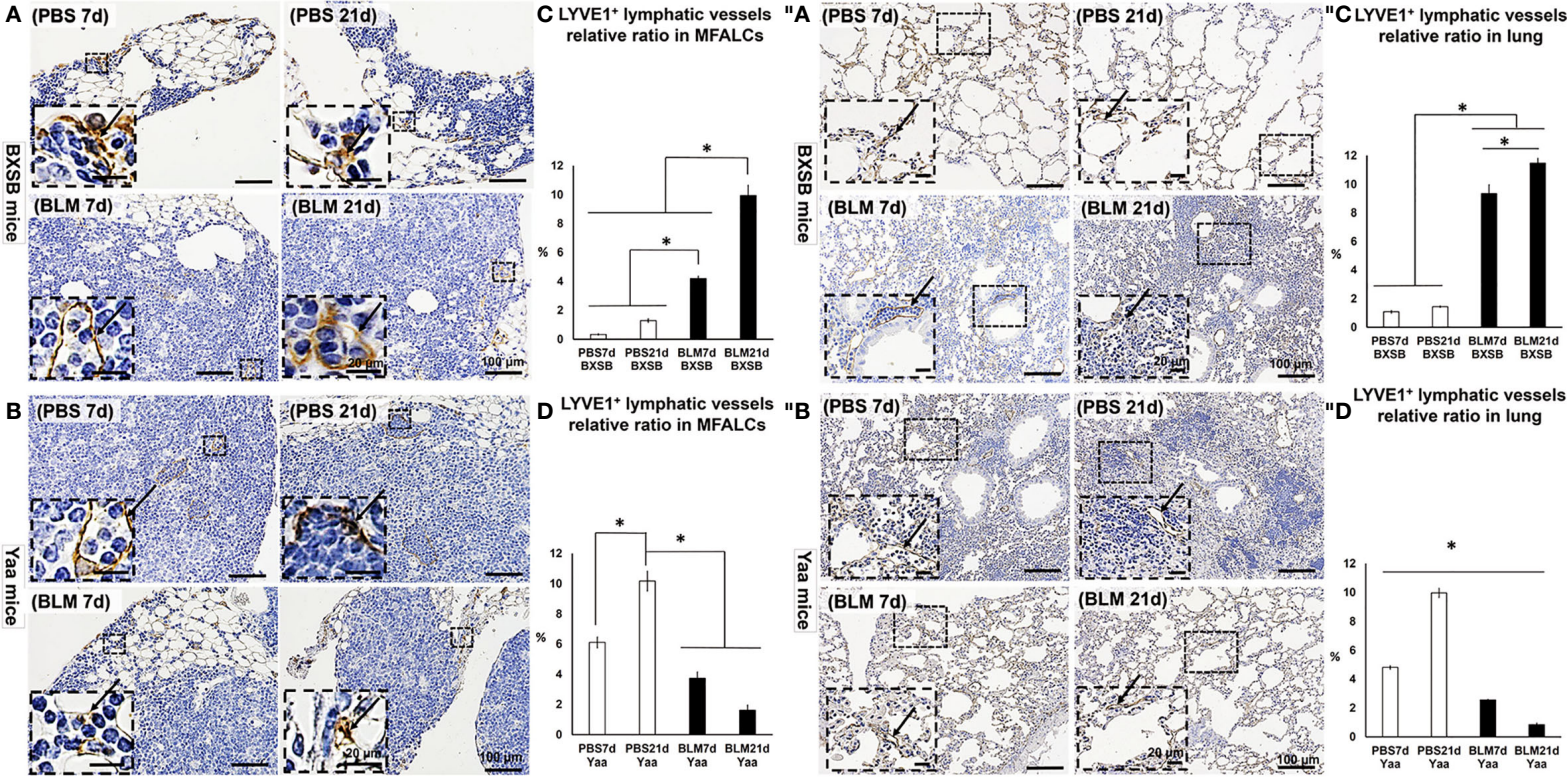
Analysis of the degree of LYVE1^+^ LV development in MFALCs **(A–D)** and lungs **(″A–″D)** of BLM and PBS groups of both autoimmune disease mice model (Yaa) and their wild-type strain (BXSB) on days 7 and 21. **(A, B)** Representative histopathological images of immunohistochemically stained MFT sections with anti-LYVE1 antibody in both BXSB **(A)** and Yaa **(B)**. **(C, D)** Graphs showing the percentages of average of relative ratios of LYVE1^+^ LVs in MFALCs of control strain “BXSB” **(C)** and its autoimmune disease mouse model “Yaa” **(D)**. **(″A, ″B)** Representative histopathological images of immunohistochemically stained lung tissue sections with anti-LYVE1 antibody in both BXSB **(″A)** and Yaa **(″B)**. Arrows indicate positive LVs. **(″C, ″D)** Graphs showing the percentages of average of relative ratios of LYVE1^+^ LVs in the control strain “BXSB” **(″C)** and its autoimmune disease mouse model “Yaa” **(″D)**. Asterisk indicates significant differences between PBS and BLM groups, analyzed by the Kruskal–Wallis test, followed by Scheffé’s method. (*P* < 0.05); *n* = 4 in each experimental group. Values are expressed as mean ± standard error (SE).

The occurrence of LVs was analyzed in MFALCs and lung tissues of both BLM and PBS groups in BXSB and Yaa mice. Furthermore, we measured the total areas of the lymphatic vessel endothelial hyaluronic acid receptor 1 (LYVE-1)^+^ LVs and the total field areas of MFALCs and the lungs. To calculate the relative ratio of LYVE1^+^ LVs areas, the total areas of LYVE-1^+^ LVs were divided by the total field areas of MFALCs and the lungs, and the average relative ratios were measured among different groups and strains. As shown in **Figures 6A, ″A**, highly developed LYVE-1^+^ LVs were observed in MFALCs (**Figure 6A**) and the lungs (**Figure 6″A**) of BXSB mice following BLM administration. The morphometrical analysis of the percentage of the relative ratio of the LYVE-1^+^ LVs areas in MFALCs and the lungs revealed a significantly increased value in the BLM group (on days 7 and 21), compared with the PBS groups in both MFALCs and the lungs. Furthermore, a significantly higher percentage was observed in the BLM group on day 21 than on day 7 (**Figures 6C, ″C**). On the contrary, less developed LYVE-1^+^ LVs were observed in the MFALCs (**Figure 6B**) and the lungs (**Figure 6″B**) of Yaa mice following BLM administration than the PBS groups, especially on day 21. Similarly, for the LVs in MFALCs and lungs, significant lower relative ratios of LYVE-1^+^ LVs were observed in the BLM group of Yaa mice on both days 7 and 21 as compared to the PBS group on day 21. Furthermore, the PBS group showed significantly higher ratios of LYVE-1^+^ LVs on day 21 than on day 7 (**Figures 6D, ″D**).

### Analysis of the Degree of Proliferation in both MFALCs and Lungs

**Figure 7:**
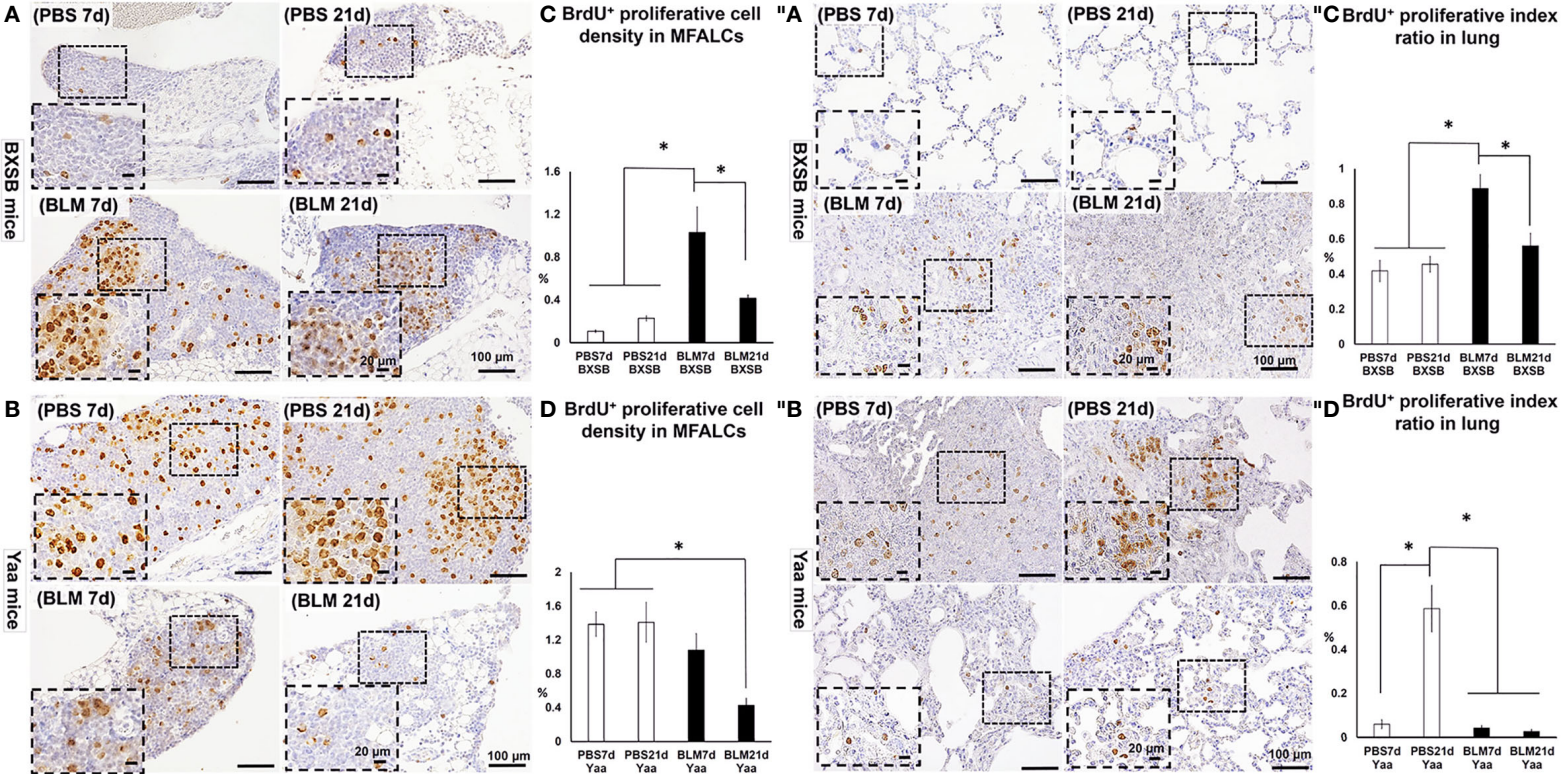
Analysis of the degree of proliferation in MFALCs **(A–D)** and lungs **(″A–″D)** of BLM and PBS groups of both autoimmune disease mice model (Yaa) and their wild-type strain (BXSB) on days 7 and 21. **(A, B)** Representative histopathological images of immunohistochemically stained MFT sections with anti-bromodeoxyuridine “BrdU” antibody in both BXSB **(A)** and Yaa **(B)**. **(C, D)** Graphs showing the percentages of BrdU^+^ proliferating cell density ratio in MFALCs of control strain “BXSB” **(C)** and its autoimmune disease mouse models “Yaa” **(D)**. **(″A, ″B)** Representative histopathological images of immunohistochemically stained lung tissue sections with anti-BrdU antibody in both BXSB **(″A)** and Yaa **(″B)**. **(″C, ″D)** Graphs showing the percentages of average of BrdU^+^ proliferating cell index ratio in control strain “BXSB” **(″C)** and its autoimmune disease mouse model “Yaa” **(″D)**. Asterisk indicates significant differences between PBS and BLM groups, analyzed by the Kruskal–Wallis test, followed by Scheffé’s method. (*P* < 0.05); *n* = 4 in each experimental group. Values are expressed as mean ± standard error (SE).

For detection of the degree of proliferation in either MFALCs or lungs, bromodeoxyuridine (BrdU)^+^ cells were observed in both BXSB and Yaa mice. Moreover, the percentage of either cell density ratio or index ratio was compared between the BLM- and PBS-treated groups in MFALCs or lungs, respectively. In BXSB mice, numerous BrdU^+^ proliferating cells were observed in MFALCs and the lungs of BLM as compared with the PBS group (**Figures 7A, ″A**). As shown in **Figures 7C, ″C**, the percentage of cell density ratio and index ratio within MFALCs and the lungs of the BLM group in BXSB mice on day 7 showed a higher percentage than those in the BLM group on day 21 and PBS group on days 7 and 21. On the contrary, the BLM groups in Yaa mice showed fewer BrdU^+^ proliferating cells, especially on day 21 as compared with the PBS groups (**Figures 7B, ″B**). The MFALCs in the BLM group on day 21 showed a significantly reduced percentage of BrdU^+^ proliferating cell density ratio than those in the PBS groups (days 7 and 21) (**Figure 7D**). Furthermore, the lungs in the PBS group on day 21 showed a significantly higher percentage of BrdU^+^ proliferating cell density ratio than those in the BLM groups (days 7 and 21) and the PBS group on day 7 (**Figure 7″D**).

### Autoimmune Indices (Spleen/BW Ratio And Serum Anti-Double Stranded DNA [anti-dsDNA] Autoantibodies)

**Figure 8:**
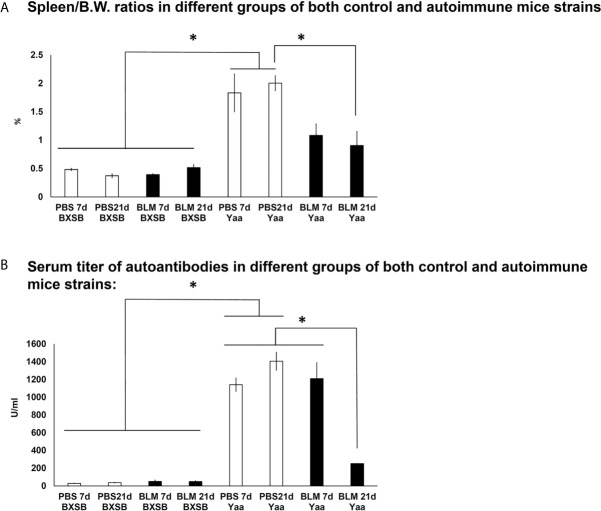
Analysis of autoimmune indices (spleen/body weight ratios and serum titer of autoantibodies) among BLM and PBS groups of both autoimmune disease mice model (Yaa) and their wild-type strain (BXSB) on days 7 and 21. **(A)** Spleen/BW ratios among BLM and PBS groups in both autoimmune disease mouse model (Yaa) and their wild-type strain (BXSB) on days 7 and 21. **(B)** Serum titer of autoantibodies among BLM and PBS groups in both autoimmune disease mouse model (Yaa) and their wild-type strain (BXSB) on days 7 and 21. Asterisk indicates significant differences between PBS and BLM groups, analyzed by the Kruskal–Wallis test, followed by Scheffé’s method. (*P* < 0.05); *n* = 4 in each experimental group. Values are expressed as mean ± standard error (SE).

To further examine the effects of BLM administration on the degree of autoimmunity, we compared the autoimmune indices among the BLM and PBS groups in both an autoimmune mouse model and their control strains. As shown in **Figure 8A**, the spleen/BW ratios of Yaa autoimmune mice were significantly higher in the PBS groups (on days 7 and 21) than in both the BLM and PBS groups (days 7 and 21) of its control (BXSB) mice strain. Furthermore, Yaa mice in the BLM group showed significantly reduced spleen/BW ratios on day 21 than the PBS group. Similarly, Yaa mice in the PBS groups (days 7 and 21) displayed significantly higher titers of serum anti-dsDNA autoantibodies than that in PBS and BLM groups in BXSB mice. Furthermore, a sharp and significant decrease in the titers of serum anti-double-stranded DNA (anti-dsDNA) autoantibodies was observed in the BLM group on day 21 than the PBS group on day 21 (**Figure 8B**).

### Relative mRNA Expression of the Yaa Locus Genes in Lung Tissues

**Figure 9:**
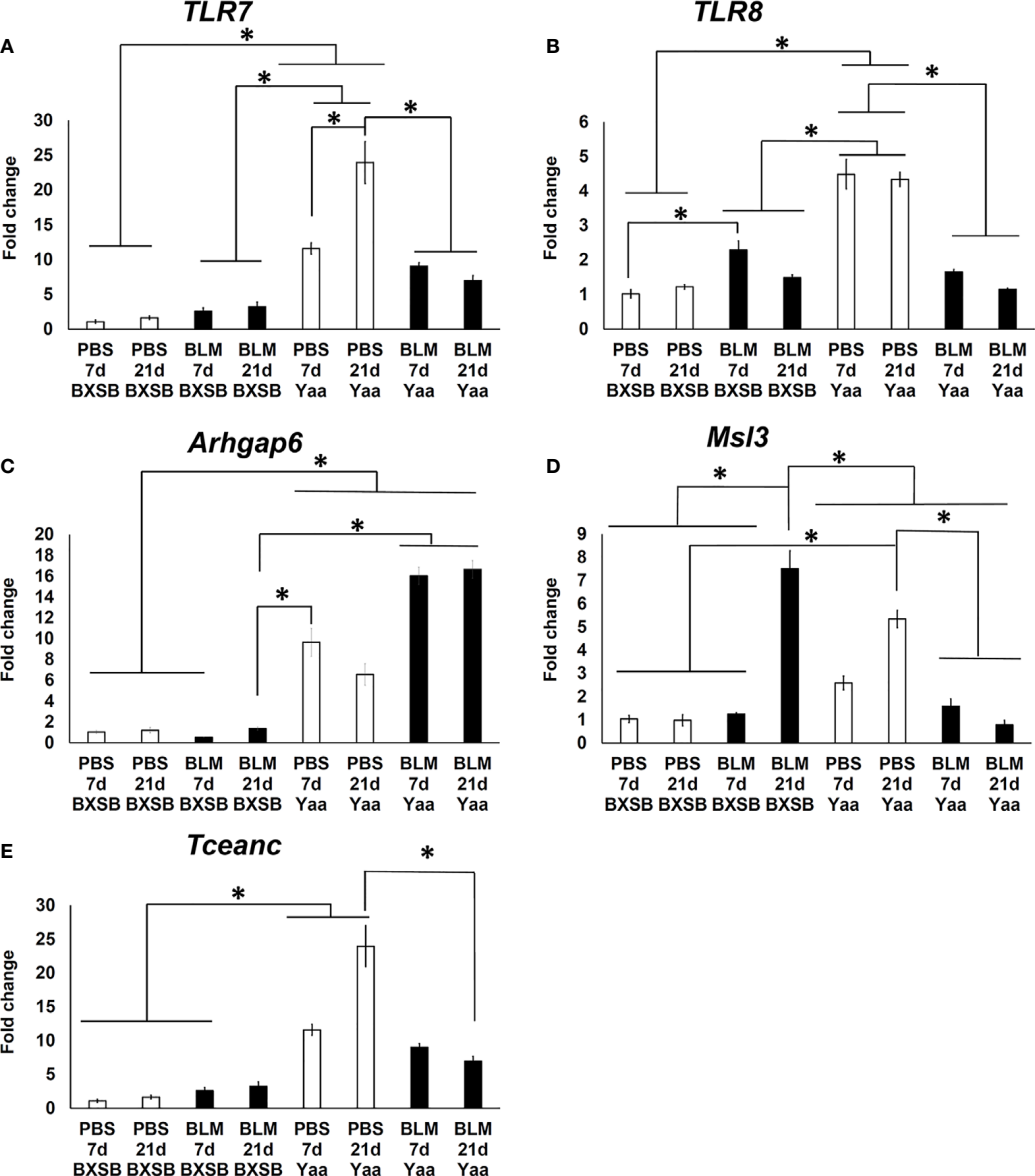
Analysis of the relative mRNA expression of Yaa locus genes (*TLR7, TLR8, Arhgap6, Msl3*, and *Tceanc)* in the lung tissues among BLM and PBS groups of both autoimmune disease mice model (Yaa) and their wild-type strain (BXSB) on days 7 and 21. Graphs showing fold increase in the relative mRNA expression of *TLR7* **(A)**, *TLR8* **(B)**, *Arhgap6* **(C)** *, Msl3* **(D)**, and *Tceanc* **(E)** in the lung tissues of different groups in both control strain “BXSB” and its autoimmune disease mouse model “Yaa.” Asterisk indicates significant differences between PBS and BLM groups, analyzed by the Kruskal–Wallis test, followed by Scheffé’s method. (*P* < 0.05); *n* = 4 in each experimental group. Values are expressed as mean ± standard error (SE).

To further explore the effect of BLM on the expression of candidate Yaa locus genes (*TLR7, TLR8, Arhgap6, Msl3*, and *Tceanc)* associated with autoimmunity, quantitative real-time PCR (RT-qPCR) was performed to compare the mRNA expression of these genes in the lung tissues of both BLM and PBS groups in Yaa and BXSB mice. Except for *Msl3*, the PBS groups (days 7 and 21) in Yaa mice showed significantly higher expression of Yaa locus genes than the BXSB mice. The PBS groups (day 21) in Yaa mice showed significantly higher expression of Msl3 mRNA than in BXSB mice (on days 7 and 21) (**Figures 9A–C** **, E**). Moreover, the BLM group on day 21 in BXSB mice revealed a significant increase in the mRNA expression of *Msl3* than the other studied groups in both BXSB and Yaa mice (**Figure 9D**). As shown in **Figures 9A, B, D**, and 9E, the BLM groups (days 7 and 21) in Yaa mice revealed a significant decrease in the mRNA expression of *TLR7, TLR8, Msl3*, and *Tceanc* than the PBS groups, especially on day 21. However, elevated mRNA expression of *Arhgap6* was observed in the BLM group of Yaa mice as compared with the PBS groups although without any significant differences (**Figure 9C**).

### Histopathological Correlations Between Autoimmune Parameters and Parameters for MFALCs and Lungs

As shown in **Tables 2A, B**, we examined the histopathological correlations between autoimmune parameters (SPW/BW, serum titer of anti-dsDNA antibodies) and parameters of MFALCs and the lungs (sizes of MFALCs, LIS, proliferating cells, and LVs and HEVs within MFALCs and the lungs) as well as between parameters of MFALCs and the lungs in both BLM and PBS groups on days 7 and 21 for autoimmune mouse model “Yaa” **Table 2A** and its control strain “BXSB” (**Table 2B**). To study correlations among different groups in Yaa mice (**Table 2A**), the SPW/BW autoimmune parameter was observed that showed a highly significant positive correlation with the size of MFALCs and BrdU^+^ proliferative cell density in MFALCs. Furthermore, the serum titer of anti-dsDNA antibodies showed a significant positive correlation with the size of MFALCs and highly significant positive correlations with the relative ratios of LYVE1^+^ LVs in MFALCs and the lungs, BrdU^+^ proliferative cell density in MFALCs, and index ratio in the lungs, and PNAd^+^ HEVs relative ratio in MFALCs. Interestingly, the size of MFALCs showed a significant positive correlation with lung BrdU^+^ proliferative cells and highly significant positive correlations with LIS and lung LVs. Furthermore, our results indicated a significant positive correlation between the quantitative indices of LVs and HEVs in MFALCs with lung HEVs and highly significant positive correlations with LIS and lung LVs. Moreover, a significant positive correlation of proliferating cells of MFALCs was observed with those of the lungs, and a highly significant positive correlation was observed with LIS and lung LVs.

**Table 2:** Spearman’s correlations between autoimmune parameters (SPW/BW, serum titer of anti-dsDNA antibodies) and the parameters of MFALCs and lungs in Yaa (1A) and BXSB (1B) mice.

Table 2A		Parameters for autoimmune disease			Parameters for MFALCs							
		SPW/BW		Anti-dsDNA Abs	MFALC	LYVE-1	PNAd	Gr1	BrdU	CD3	B220	Iba1
**Parameters for autoimmune disease in Yaa**	**SPW/BW**	*ρ*	–	0.179	0.632**	0.412	0.235	-0.047	0.641**	-0.056	-0.541*	0.424
		*P*	–	0.506	0.009	0.113	0.113	0.863	0.007	0.837	0.030	0.102
	**Anti-dsDNA Abs**	*ρ*	0.179	–	0.603*	0.765**	0.771**	-0.150	0.644**	0.344	-0.256	-0.344
		*P*	0.506	–	0.013	0.001	0.001	0.579	0.007	0.192	0.339	0.192
**Parameters for lung in Yaa**	**LIS**	*ρ*	0.494	0.390	0.868**	0.806**	0.711**	0.475	0.695**	-0.007	-0.604*	-0.669**
		*P*	0.052	0.136	0.001	0.001	0.002	0.063	0.003	0.978	0.013	0.005
	**BrdU**	*ρ*	0.188	0.641**	0.509*	0.762**	0.756**	0.050	0.544*	0.282	-0.332	-0.553*
		*P*	0.485	0.007	0.044	0.001	0.001	0.854	0.029	0.289	0.208	0.026
	**Gr1**	*ρ*	0.641**	0.009	0.350	0.162	0.074	0.053	0.365	-0.053	-0.476	-0.171
		*P*	0.007	0.974	0.184	0.549	0.787	0.846	0.165	0.846	0.062	0.528
	**CD3**	*ρ*	0.238	0.700**	0.697**	0.815**	0.811**	0.350	0.697**	0.279	-0.444	-0.465
		*P*	0.374	0.003	0.003	0.001	0.001	0.184	0.004	0.295	0.085	0.070
	**B220**	*ρ*	0.444	0.285	0.632**	0.724**	0.729**	0.509*	0.538*	-0.132	-0.694**	-0.803**
		*P*	0.085	0.284	0.009	0.002	0.003	0.044	0.031	0.625	0.003	0.001
	**Iba1**	*ρ*	0.365	0.474	0.691**	0.782**	0.768**	0.456	0.556*	0.006	-0.618*	-0.674**
		*P*	0.165	0.064	0.003	0.001	0.001	0.076	0.025	0.983	0.011	0.004
	**LYVE-1**	*ρ*	0.424	0.674**	0.874**	0.947**	0.911**	0.247	0.788**	0.159	-0.582*	-0.671**
		*P*	0.102	0.004	0.001	0.001	0.001	0.356	0.001	0.557	0.018	0.004
	**PNAd**	*ρ*	0.286	0.182	0.497	0.522*	0.544*	0.247	0.278	0.007	-0.478	-0.425
		*P*	0.284	0.500	0.050	0.038	0.029	0.356	0.297	0.978	0.061	0.101

LIS, lung Injury Score. MFALC: % of LCs area/total MFTs area. P: Spearman’s rank correlation coefficient. n= 4/group (PBS 7, 21 d and bleomycin7, 21 d. *: Significant, P <0.05. **: highly significant, P < 0.01.

**Table:** 

Table 2B		Parameters for autoimmune disease			Parameters for MFALCs							
		SPW/BW		Anti-dsDNA Abs	MFALC	LYVE-1	PNAd	Gr1	BrdU	CD3	B220	Iba1
**Parameters for autoimmune disease in BXSB**	**SPW/BW**	*ρ*	–	-0.132	0.085	0.150	0.053	0.253	0.459	-0.256	0.247	0.118
		*P*	–	0.625	0.753	0.579	0.846	0.345	0.074	0.339	0.356	0.664
	**Anti-dsDNA Abs**	*ρ*	-0.132	–	0.388	0.391	0.409	0.444	0.459	0.329	0.262	-0.094
		*P*	0.625	–	0.137	0.213	0.116	0.085	0.074	0.213	0.327	0.729
**Parameters for lung in BXSB**	**LIS**	*ρ*	0.396	0.387	0.709**	0.876**	0.837**	0.711**	0.637**	0.620*	0.815**	0.165
		*P*	0.129	0.139	0.002	0.001	0.001	0.002	0.008	0.010	0.001	0.542
	**BrdU**	*ρ*	-0.135	0.179	0.529*	0.412	0.497	0.129	0.774**	0.747**	0.271	0.209
		*P*	0.617	0.506	0.035	0.113	0.050	0.633	0.001	0.001	0.311	0.438
	**Gr1**	*ρ*	0.106	0.450	0.768**	0.838**	0.712**	0.350	0.809**	0.729**	0.591*	0.341
		*P*	0.696	0.080	0.001	0.001	0.002	0.184	0.001	0.001	0.016	0.196
	**CD3**	*ρ*	-0.215	0.379	0.683**	0.706**	0.606*	0.315	0.853**	0.750**	0.538*	0.203
		*P*	0.425	0.147	0.008	0.002	0.013	0.235	0.001	0.001	0.031	0.451
	**B220**	*ρ*	-0.103	0.238	0.685**	0.556*	0.688**	0.421	0.818**	0.803**	0.406	0.182
		*P*	0.704	0.374	0.003	0.025	0.003	0.105	0.001	0.001	0.119	0.499
	**Iba1**	*ρ*	-0.218	0.432	0.624**	0.738**	0.676**	0.379	0.935**	0.856**	0.559*	0.200
		*P*	0.418	0.094	0.010	0.001	0.004	0.147	0.001	0.001	0.024	0.458
	**LYVE-1**	*ρ*	0.176	0.379	0.750**	0.953**	0.909**	0.644**	0.765**	0.741**	0.750**	0.362
		*P*	0.513	0.147	0.001	0.001	0.001	0.007	0.001	0.001	0.001	0.169
	**PNAd**	*ρ*	0.007	0.495	0.782**	0.478	0.475	0.301	0.760**	0.755**	0.441	0.115
		*P*	0.980	0.051	0.001	0.061	0.063	0.258	0.001	0.001	0.087	0.672

LIS, lung Injury Score. MFALC: % of LCs area/total MFTs area. ρ: Spearman’s rank order correlation coefficient. n= 4/group (PBS 7, 21 d &bleomycin7, 21 d. *: Significant, P < 0.05. **: highly significant, P < 0.01.

In addition, we examined the correlations between different parameters of MFALCs and the lungs in the control strain (BXSB). As shown in **Table 2B**, the LIS showed highly significant correlations with the size of MFALCs, and both the quantitative indices of LV and HEVs. In addition, the quantitative indices of LV and HEVs in the lungs revealed a highly significant positive correlation with those of the MFALCs as well as with the size of MFALCs.

## Discussion

### Effect of BLM Administration on the Degree of Lung Injury and MFALC Development and Autoimmune Mice Model

Systemic autoimmune disorders constitute a group of immune-mediated inflammatory diseases affecting various organs, including the lungs, heart, kidneys, skin, brain, and hematopoietic system. They are characterized by the deposition of immune complexes or direct autoantibodies resulting in tissue inflammation and damage (22, 23). Several autoimmune diseases (AID) have been reported in humans, including SLE, systemic sclerosis, rheumatoid arthritis (RA), polymyositis/dermatomyositis, Wegener’s granulomatosis, ankylosing spondylitis, and Sjögren syndrome (23). Recently, several autoimmune murine strains have been used as models to study the pathogenesis of AID development in humans, such as Lpr and male Yaa mice. These mice displayed lesions that closely mimicked human AID, especially in SLE and RA, including lymphadenopathy, splenomegaly, and hypergammaglobulinemia with anti-dsDNA antibodies (13, 24). Furthermore, pleuropulmonary involvement and the development of lung injury have been reported to occur in most AID in both humans and mouse models (6, 23, 25, 26).

Interestingly, we previously reported the presence of more prominent MFALCs in autoimmune disease mouse models (Lpr and Yaa mice) as compared with control strains. The study showed a significant positive correlation between the size of MFALCs and cellular infiltration in the lungs of these models as well as with autoimmune disease indices, including the spleen/BW ratios and serum autoantibody levels, suggesting a possible function of MFALCs in the progression of lung lesions (6). Furthermore, previous studies have demonstrated that inflammation induced the development of FALCs in the mesentery and MFTs following peritoneal immunization with antigens and intranasal (i.n.) BLM administration, respectively (3, 5). In humans, BLM has been previously used as a successful therapeutic target for certain curable malignancies, such as germ cell tumors, and Hodgkin’s and non-Hodgkin’s lymphomas. However, its use is currently not recommended due to the development of pulmonary fibrosis in up to 10% of patients receiving the medication (9, 27). Altogether, differences in mouse strain have been reported to be susceptible to the development of pulmonary fibrosis following BLM administration. Specifically, A/J and Balb/c mice were found to be resistant to fibrosis, whereas both DBA and B6 mice were highly susceptible (11, 28). Furthermore, our recent study had demonstrated that BLM induced the development of pneumonitis in healthy B6 mice, concomitantly with an increased size of MFALCs when compared with the PBS-administered control group (7). However, the effect of BLM on the degree of lung injury and MFALCs remain largely unclear in Yaa autoimmune disease mouse models, characterized by lymphoproliferation and more developed MFALCs. Therefore, we examined the effect of BLM on the degree of lung injury in autoimmune disease mouse models (Yaa) and their corresponding control strain (BXSB) on days 7 and 21 following i.n. administration of either BLM sulfate (5 mg/kg) or vehicles (PBS group). The present study revealed a reduced degree of lung injury and size of MFALCs following BLM administration in Yaa mice as compared with the corresponding PBS group; however, contradictory results were observed in their wild-type control strain. These results suggest a dual effect of BLM on lung injury and intrathoracic immune hemostasis that could be attributed to strain differences in response to BLM administration.

### Effect of BLM Administration on Immune Cells’ Hemostasis in Both Lungs and MFALCs in the Autoimmune Mice Model

FALCs are immune cell aggregates that associate with mediastinal and mesenteric fat tissues. Both lineage-positive (B and T lymphocytes) and lineage-negative (macrophages and granulocyte) cells are enriched with FALCs (1, 2). The present investigation revealed a significant positive correlation between the size of MFALCs and the degree of lung injury in all analyzed groups, suggesting the possible function of MFALCs in immune cell recruitment into the lung tissues. Furthermore, we analyzed the positive area ratios for immune cells within both MFALCs and lung tissues among the BLM and PBS groups in all analyzed strains. Interestingly, the BLM group in BXSB mice showed significantly higher ratios for B and T lymphocytes, and macrophages and a higher tendency for granulocytes than the PBS group in both MFALCs and the lungs. However, a significantly lower positive area ratio for B and T lymphocytes and macrophages was observed in the lungs of Yaa mice in the BLM group as compared with the PBS control group. Furthermore, the ratios of B and T lymphocytes and macrophages within MFALCs were observed to be negatively and positively correlated with the degree of lung injury in Yaa and BXSB, respectively. Therefore, based on our findings, we suggest that BLM could exert different effects on the recruitment of immune cells into the lung tissues among various mouse strains, and thus a dual effect of BLM was observed on lung fibrosis. Consistent with our results showing a decrease in the quantitative indices of T and B lymphocytes, and macrophages in the lung tissues of Yaa mice following BLM administration, previous reports revealed the function of different immune cells in the progression of pulmonary fibrosis (29). Our results revealed a reduced ratio of T lymphocytes within MFALCs and the lungs of the BLM group on day 21 than on day 7 in both studied strains. Interestingly, a recent study demonstrated that infiltration of T lymphocytes in the lungs contributed to the progression of pulmonary fibrosis through a profibrotic function or recovery from fibrosis through an antifibrotic role (30). In addition, a dual effect of B lymphocytes on the progression of pulmonary fibrosis in a mouse model of BLM-induced fibrosis was reported where the progression of lung fibrosis was substantially exacerbated when B cell depletion occurred before inducing lung fibrosis lesions. However, this fibrosis was dramatically suppressed when B cell depletion occurred during the inflammatory phase (31).

### Effect of BLM Administration on the Degree of Proliferation, Development of LVs, and HEVs in Both Lungs and MFALCs in the Autoimmune Mice Model and Its Control Wild Strain

Previous studies have implicated inflammation induced by chemicals or helminth or bacterial infections in the rapid formation and expansion of FALCs by triggering the proliferation of immune cells (1, 3, 5). Similarly, our recent study revealed a significant increase in the ratio of the proliferation of immune cells as well as the ratio of LV areas in both lungs and MFALCs following BLM administration, suggesting its function in the development of lung injury (7). Consistent with this speculation, we demonstrated a significant increase in the degree of proliferation (BrdU^+^ proliferative cell density) and the relative ratio of LYVE1^+^ LVs in both MFALCs and the lungs of the BLM group as compared with the PBS group in BXSB mice. However, an opposite trend was observed in the case of Yaa mice.

HEVs are specialized blood vessels that play a vital role in lymphocyte trafficking. HEVs were first reported in secondary lymphoid organs, such as lymph nodes (LN) and Peyer’s patches of healthy individuals (32). A recent study reported that HEVs can develop during chronic inflammation associated with autoimmunity, allografts, and infection in non-lymphoid organs; thus, playing a significant function in the recruitment of lymphocytes at the inflamed sites (33). Furthermore, our recent study showed the development of HEVs and LVs in the lung tissues of B6 mice following BLM administration but not following PBS treatment, suggesting their function in the development of lung injury (7). Interestingly, the present study revealed higher quantitative values for HEVs within MFALCs of the BLM groups of the wild-type control BXSB mice and lower values in Yaa mice as compared with the PBS control groups. Furthermore, we demonstrated the presence of well-developed HEVs in the lungs of BLM groups in the control strain (BXSB mice); however, these were not detected in the PBS groups. Furthermore, less-developed HEVs were observed in the BLM group of Yaa mice in comparison with the corresponding PBS group. Therefore, our data suggest a possible function of HEVs in the development of lung injury. In addition, our study suggests that the dual effect of BLM on lung injury development could be related to their effect on HEV development. Therefore, HEVs may be considered as therapeutic targets for lung injuries associated with autoimmune diseases. However, further studies are required to explore other possible mechanisms and associated factors beyond the development of HEVs in the lungs following BLM administration, especially in the control strains.

### Effect of BLM Administration on the Degree of Autoimmunity, Relative Expression of Both Candidate Fibrosis and Yaa Locus Genes in the Autoimmune Mice Model and Its Control Wild Strain

The present study revealed a dramatic decrease in the autoimmune indices (spleen/BW ratios and serum titers of anti-dsDNA autoantibodies) on day 21 following BLM administration in the Yaa autoimmune mouse model, suggesting reduced severity of autoimmunity-associated lesions. However, no significant change in such indices among BXSB groups was observed. Furthermore, we revealed a significant increase in candidate fibrosis genes (*Acta2, Col1a1*, and *Col3a1*) in the BLM groups of BXSB mice on day 21 than other groups in both BXSB and Yaa mice. However, reduced expression of such genes was observed following BLM administration in Yaa, as compared with the PBS groups. Consistent with our results, the role of different mouse strains in the susceptibility to BLM-induced pulmonary fibrosis have been reported (10, 11). Moreover, previous studies have reported that changes in the expression of certain genes in the lung tissues could enhance the progression of lung fibrosis (11, 28). Recently, it has been reported that the susceptibility of B6 mice to pulmonary fibrosis following BLM administration was linked to the major locus on chromosome 17, called BLM-induced pulmonary fibrosis 1 (*Blmpf1*) (11). For Yaa locus genes in the autoimmune mouse model (Yaa mice), the autoimmunity was observed only in the males due to translocation of a region of the X chromosome onto the Y chromosome, leading to the duplication of 16 genes on the Y chromosome (34, 35). Interestingly, in our recent study, we revealed *TLR7, TLR8, Arhgap6, Msl3*, and *Tceanc* as major candidate Yaa-associated locus genes associated with promoting severe autoimmune response with an increase in age (18). Furthermore, these genes play a vital role in the progression of various lung diseases, including chronic obstructive pulmonary diseases, idiopathic interstitial pneumonia, lung injury, and lung cancer (35–38). Nevertheless, the effect of BLM administration on the expression of Yaa locus genes has not been reported that could contribute to the diverse effects of BLM on the degree of lung injury among the autoimmune Yaa mouse model and its corresponding strain. Therefore, we compared the expression of these genes in the lung tissues of Yaa and BXSB mice in both BLM and PBS groups. Except for *Arhgap6*, our results revealed significant downregulation in the expression of other studied genes in the BLM group of Yaa mice as compared with the PBS groups, especially on day 21. In contrast, elevated relative expression of *Arhgap6* was observed in the BLM group of Yaa mice than in the PBS group. The upregulation of *Arhgap6* gene is critically important in preventing and treating lung cancer through the suppression of matrix metalloproteinase−9 (MMP9) and vascular endothelial growth factor (VEGF) (36). Interestingly, the inhibition of TLR-7 in experimental lupus attenuated glomerulonephritis and lung injury (39). Alveolar macrophages express higher levels of TLR1, TLR2, TLR4, TLR7, and TLR8 (40). The current study indicated a significant decrease in lung macrophages and in the relative expression of both TLR7 and TLR8 in Yaa mice following BLM administration when compared with PBS groups. In addition, the expression of *Msl3* gene was higher by 85.2 fold in patients with idiopathic interstitial pneumonia (IIP) when compared with the control (38). Similarly, the BLM group in BXSB mice on day 21 showed significant upregulation of the latter gene than other groups in BXSB and Yaa mice. In contrast, the BLM groups (days 7 and 21) in Yaa mice showed significant downregulation of *Msl3* gene expression than the PBS group, as well as the BLM group (day 21) in BXSB mice. Thus, our results suggested that the upregulation of such genes contributed to a higher degree of lung fibrosis progression observed in BXSB on day 21 following BLM administration than other studied groups.

## Conclusions

The present study sheds light on strain-related differences following BLM administration in an autoimmune mouse model (Yaa) and its control strain (BXSB). BLM dramatically increased and decreased lung injury in BXSB and Yaa mice, respectively. Our results described multifactorial roles of BLM, which could explain its ameliorative effect on the degree of lung injury in Yaa mice, including downregulation of certain Yaa locus genes as well as its suppressive effect on HEV development, proliferation activity of immune cells, and their recruitment activity into the lung tissue, especially for macrophages, and T and B lymphocytes. Therefore, the present study suggested that targeting these factors could be a novel strategy for therapeutic approaches for lung injury associated with autoimmune diseases.

## Data Availability Statement

The original contributions presented in the study are included in the article/**Supplementary Material**. Further inquiries can be directed to the corresponding author.

## Ethics Statement

The animal study was reviewed and approved by Institutional Animal Care and Use Committee of the Graduate School of Veterinary Medicine, Hokkaido University (approval no. 15–0079).

## Author Contributions

YE designed the study and experiments, interpreted the data, conceived the article structure, and wrote the manuscript. OI designed the experiments and edited the manuscript. TN reviewed the manuscript. YK conceptualized the research, performed data interpretation, contributed to the discussion, and critically revised the manuscript draft. All authors contributed to the article and approved the submitted version.

## Funding

This study was supported by the Hokkaido University Tenure Track Program (Research Support Program II for Young Researchers) and Grant-in-Aid for Scientific Research (KAKENHI “B” No. 19H03113 and KAKENHI “C” No. 20K07420).

## Conflict of Interest

The authors declare that the research was conducted in the absence of any commercial or financial relationships that could be construed as a potential conflict of interest.

## Supplementary Material

Supplementary Figure 1 Comparison between the lung injury score of BLM and PBS groups in both autoimmune disease mice model (Yaa) and their wild-type strain (BXSB) and the wild-type strain (B6 mice) on days 7 and 21. Graphs showing the average of lung injury score among studied groups and the wild-type strain. Asterisk indicates significant difference, analyzed by the Kruskal–Wallis test, followed by Scheffé’s method. (*P* < 0.05); *n* = 4 in each experimental group. Values are expressed as mean± standard error (SE).

Supplementary Figure 2 Collagen fibers (collagen I, col3A1) deposition in the lung sections of BLM and PBS groups in both autoimmune disease mice model (Yaa) and their wild-type strain (BXSB) on days 7 and 21. **(A, B)** Representative lung sections immuno-stained with anti-collagen I in both BXSB **(A)** and Yaa **(B)**. **(D, E)** Representative lung sections immuno-stained with anti-col3A1 in both BXSB **(D)** and Yaa **(E)**. **(C, F)** Graphs showing the percentages of positive area ratios of collagen I **(C)**, and col3A1 **(F)** among studied groups. Asterisk indicates significant differences between PBS and BLM groups, analyzed by the Kruskal–Wallis test, followed by Scheffé’s method. (*P* < 0.05); *n* = 4 in each experimental group. Values are expressed as mean± standard error (SE).

Supplementary Figure 3 Double immunofluorescent staining of MFALCs and lung sections of BLM and PBS groups in both autoimmune disease mice model (Yaa) and their wild-type strain (BXSB) on days 7 and 21. **(A, B)** Representative immunofluorescent images of MFALCs stained with anti-B220 and anti-CD3 antibodies in both BXSB **(A)** and Yaa **(B)**. **(C, D)** Representative immunofluorescent images of lung sections stained with anti-B220 and anti-CD3 antibodies in both BXSB **(C)** and Yaa **(D)**. Notice B220^+^ B cells (green) and CD3^+^ T cells (red).

Supplementary Figure 4 Immunohistochemical staining of macrophages in MFALCs and the lungs of BLM and PBS groups in both autoimmune disease mice model (Yaa) and their wild-type strain (BXSB) on days 7 and 21. **(A, B)** Representative histopathological images of immunohistochemically stained sections of MFALCs in both BXSB **(A)** and Yaa **(B)** and the lungs in both BXSB **(C)** and Yaa **(D)** with anti-Iba1 antibody.
