# Supplementary figures and images for: Dual Effect of Bleomycin on Histopathological Features of Lungs and Mediastinal Fat-Associated Lymphoid Clusters in an Autoimmune Disease Mouse Model
Source: Front Immunol. 2021 Jul 21;12:665100. doi: 10.3389/fimmu.2021.665100 (PMC8335540; doi:10.3389/fimmu.2021.665100)

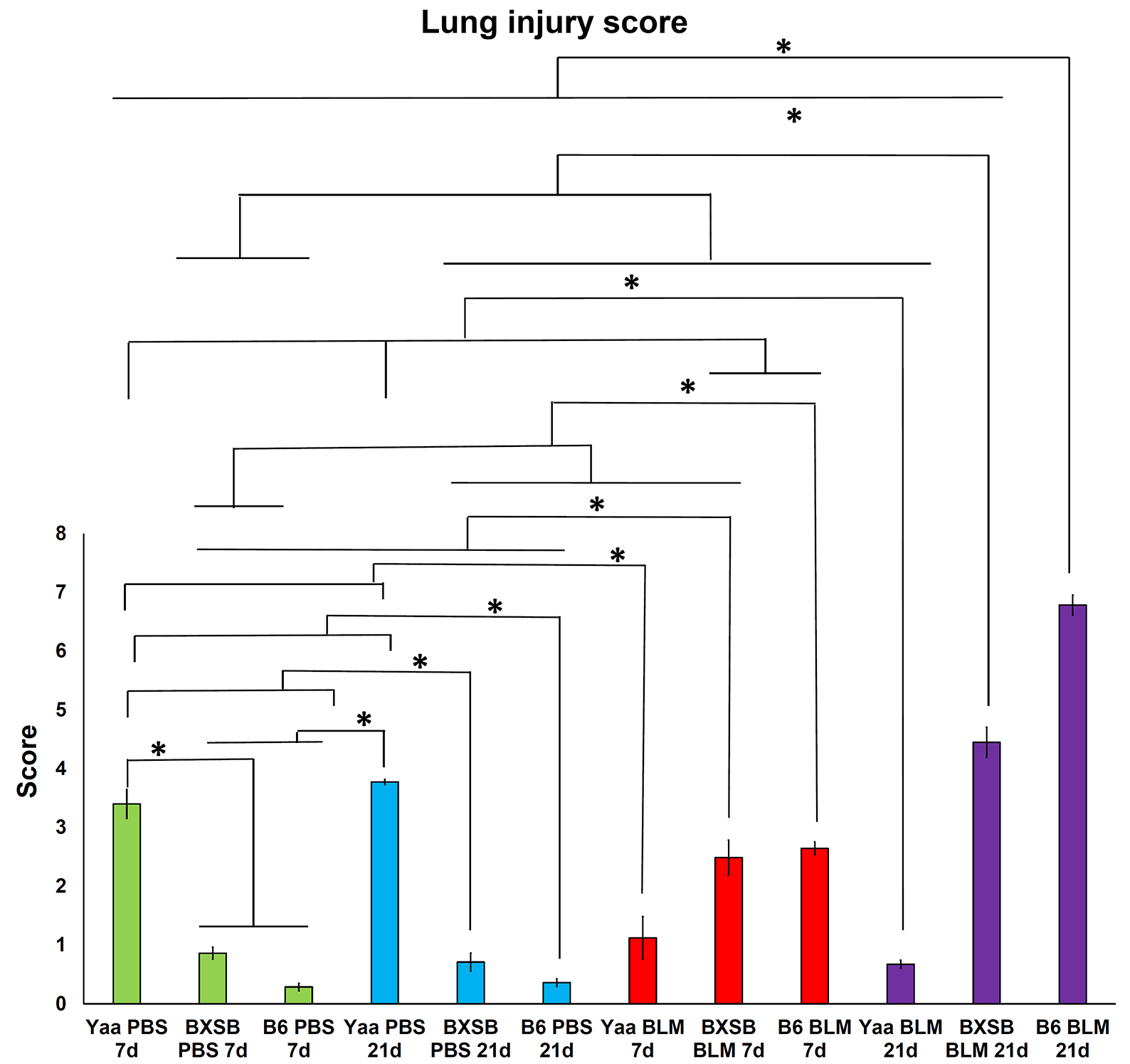

Supplement: Supplementary Figure 1 — Comparison between the lung injury score of BLM and PBS groups in both autoimmune disease mice model (Yaa) and their wild-type strain (BXSB) and the wild-type strain (B6 mice) on days 7 and 21. Graphs showing the average of lung injury score among studied groups and the wild-type strain. Asterisk indicates significant difference, analyzed by the Kruskal–Wallis test, followed by Scheffé’s method. (P < 0.05); n = 4 in each experimental group. Values are expressed as mean± standard error (SE). [file Image_1.tif]

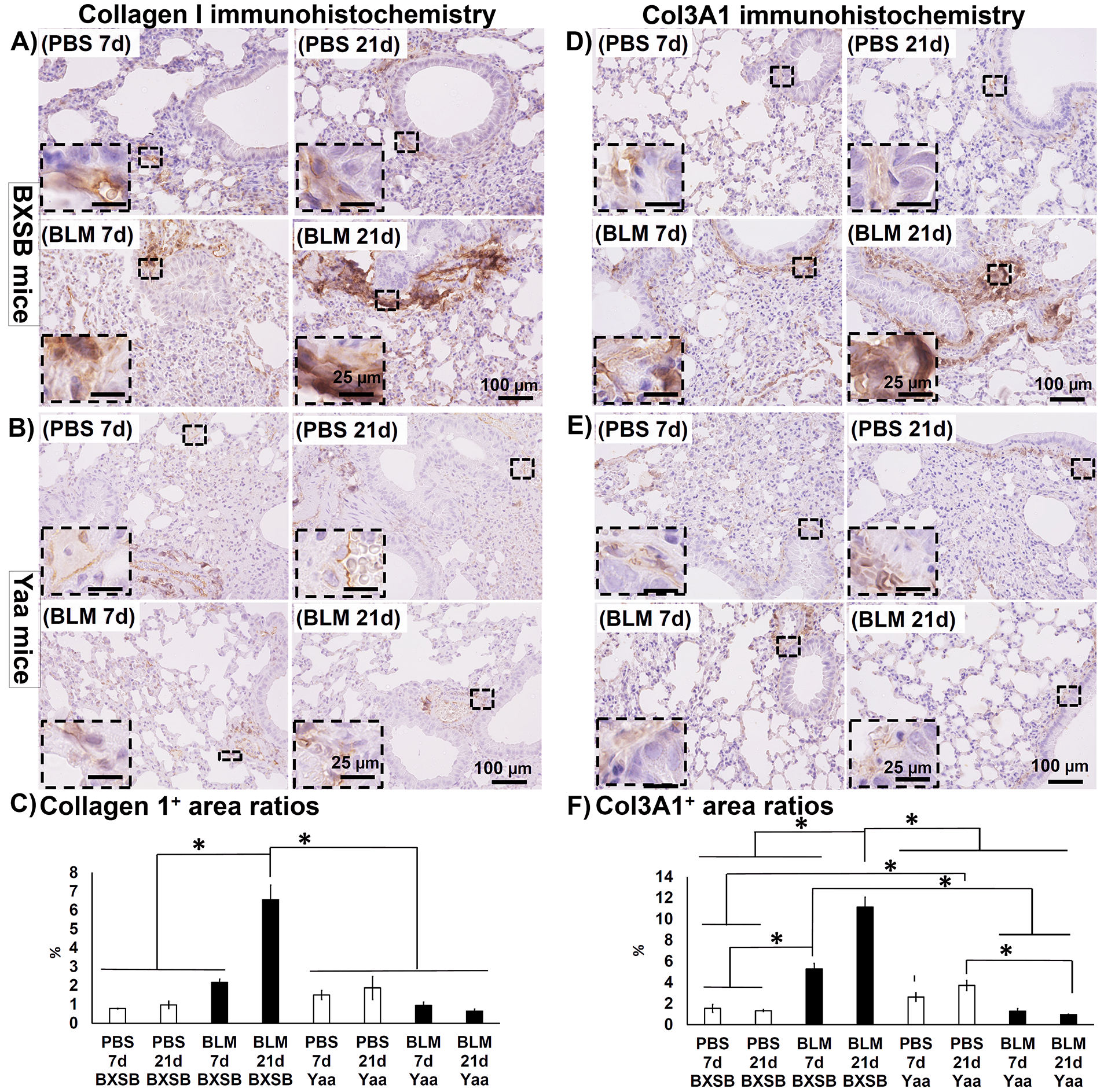

Supplement: Supplementary Figure 2 — Collagen fibers (collagen I, col3A1) deposition in the lung sections of BLM and PBS groups in both autoimmune disease mice model (Yaa) and their wild-type strain (BXSB) on days 7 and 21. (A, B) Representative lung sections immuno-stained with anti-collagen I in both BXSB (A) and Yaa (B). (D, E) Representative lung sections immuno-stained with anti-col3A1 in both BXSB (D) and Yaa (E). (C, F) Graphs showing the percentages of positive area ratios of collagen I (C), and col3A1 (F) among studied groups. Asterisk indicates significant differences between PBS and BLM groups, analyzed by the Kruskal–Wallis test, followed by Scheffé’s method. (P < 0.05); n = 4 in each experimental group. Values are expressed as mean± standard error (SE). [file Image_2.tif]

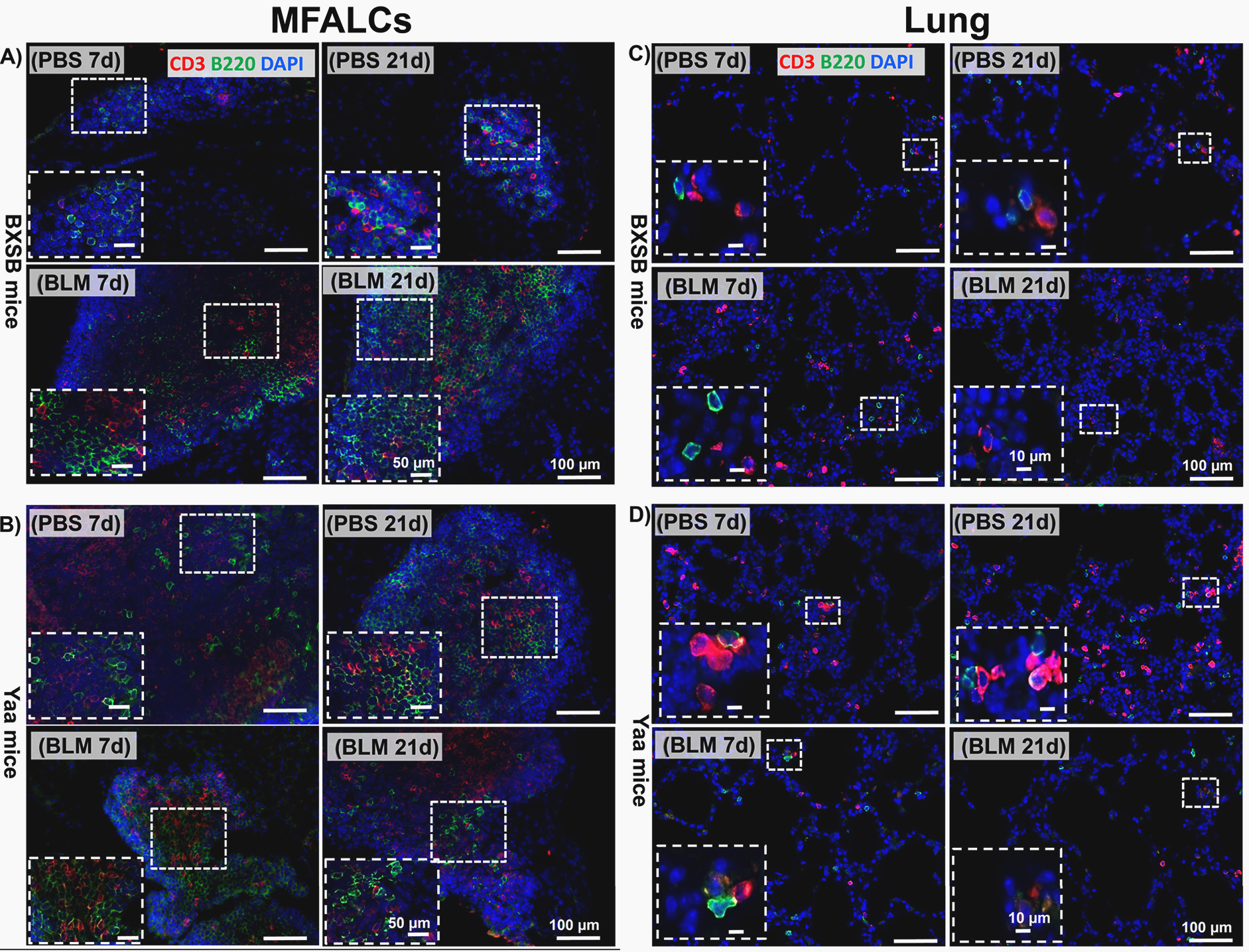

Supplement: Supplementary Figure 3 — Double immunofluorescent staining of MFALCs and lung sections of BLM and PBS groups in both autoimmune disease mice model (Yaa) and their wild-type strain (BXSB) on days 7 and 21. (A, B) Representative immunofluorescent images of MFALCs stained with anti-B220 and anti-CD3 antibodies in both BXSB (A) and Yaa (B). (C, D) Representative immunofluorescent images of lung sections stained with anti-B220 and anti-CD3 antibodies in both BXSB (C) and Yaa (D). Notice B220+ B cells (green) and CD3+ T cells (red). [file Image_3.tif]

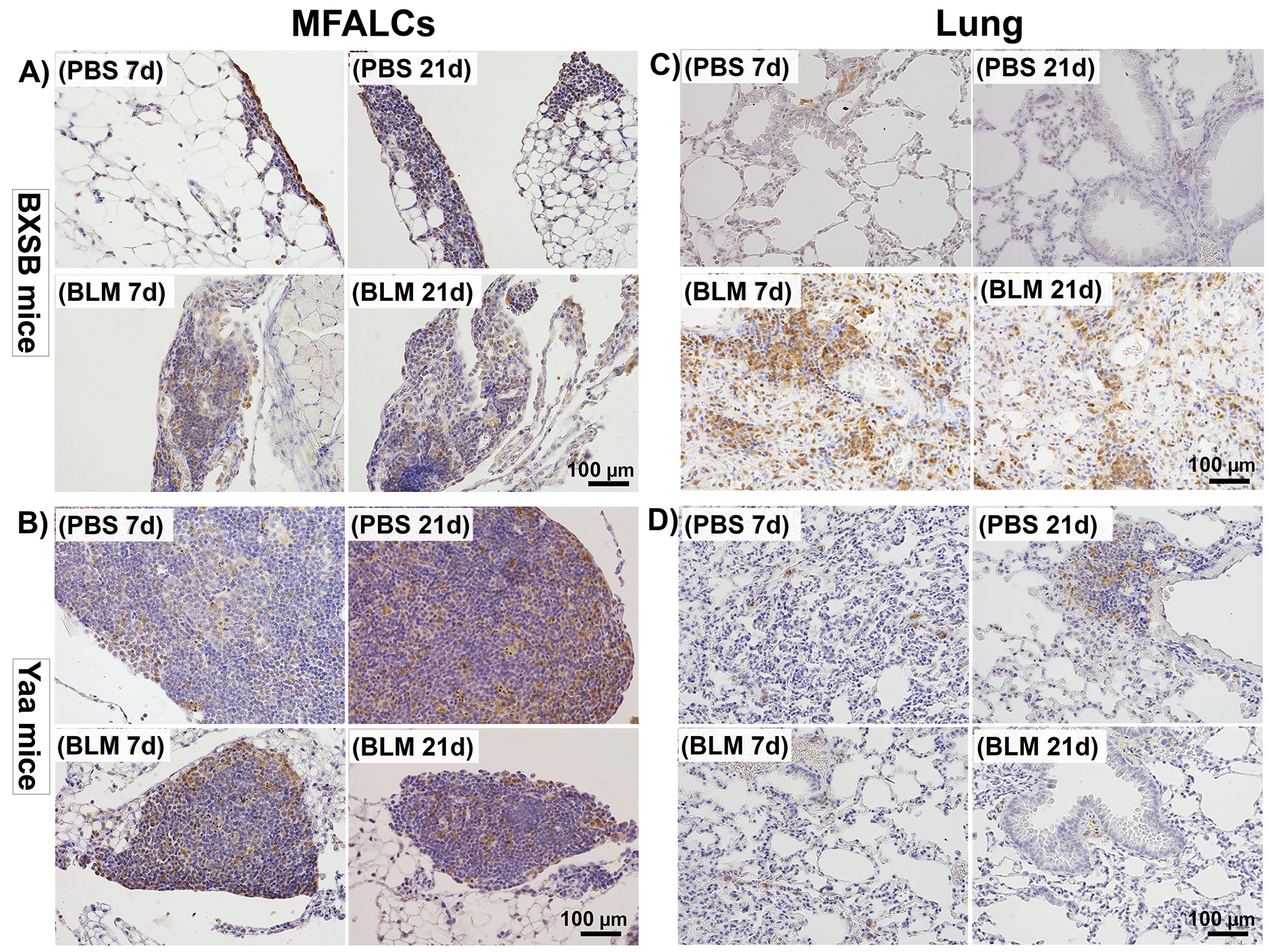

Supplement: Supplementary Figure 4 — Immunohistochemical staining of macrophages in MFALCs and the lungs of BLM and PBS groups in both autoimmune disease mice model (Yaa) and their wild-type strain (BXSB) on days 7 and 21. (A, B) Representative histopathological images of immunohistochemically stained sections of MFALCs in both BXSB (A) and Yaa (B) and the lungs in both BXSB (C) and Yaa (D) with anti-Iba1 antibody. [file Image_4.tif]
